# Quality Markers of *Dendrobium officinale* by “Oligosaccharide-Spectrum-Effect” Relationships

**DOI:** 10.3389/fnut.2022.914380

**Published:** 2022-06-06

**Authors:** Ruimin Liu, Songshan Shi, Si Xiong, Juan Su, Xiaona Gan, Jianjun Wu, Huijun Wang, Shunchun Wang

**Affiliations:** ^1^The MOE Key Laboratory for Standardization of Chinese Medicines and the SATCM Key Laboratory for New Resources and Quality Evaluation of Chinese Medicines, Institute of Chinese Materia Medica, Shanghai University of Traditional Chinese Medicine, Shanghai, China; ^2^Nutrilite Health Institute, Amway (China) Co., Ltd., R&D Center, Shanghai, China

**Keywords:** *Dendrobium officinale*, oligosaccharides, anti-inflammatory, spectrum-effect relationships, quality markers (Q-markers)

## Abstract

*Dendrobium officinale* Kimura et Migo has been used as a traditional Chinese medicine (TCM) and a functional food for thousands of years. Carbohydrate is one of the most important effective substances and indicative components in *D. officinale*. However, since the qualitative and quantitative analysis of polysaccharides in *D. officinale* remains a challenge and limitation, herein, an oligosaccharide-quality marker approach was newly developed for quality assessment of *D. officinale* by spectrum–effect relationships between high performance liquid chromatographic (HPLC) fingerprints and anti-inflammatory effects. The HPLC fingerprints of 48 batches of oligosaccharides from *D. officinale* (DOOS) were developed and analyzed with similarity analysis (SA) and hierarchical cluster analysis (HCA), and eight common peaks were identified. *In vitro* screening experiment indicated that DOOS potentially inhibited nitric oxide (NO) production and effectively reduced the release of inflammatory cytokines, such as TNF-α, IL-6, and IL-1β in RAW 264.7 cells, thereby reducing the inflammatory response of cells. Finally, the HPLC fingerprint of different batches of DOOS was combined with *in vitro* anti-inflammatory activity to assess the spectrum–effect relationships of DOOS by gray correlation analysis (GCA), in addition, the purified oligosaccharide components were identified and validated for NO inhibitory activity. Our results showed four DOOS (maltotetraose, maltopentaose, maltohexaose, and mannohexaose) were relevant to anti-inflammatory effects and could be as quality markers for the quality control of *D. officinale*. It suggests that the “oligosaccharide-spectrum-effect” relationships approach is a simple and reliable method for the quality control of herb medicines or nutritious foods.

## Introduction

*Dendrobium* is one of the largest genera of orchids. More than 1,100 species of *Dendrobium* have been identified worldwide. A total of 74 species are found in China, of which more than 50 species can be used as China’s precious *Dendrobium* Tea. *Dendrobium officinale* Kimura et Migo, Chinese name Tiepi Shihu, is a perennial herb in the biological classification of Orchidaceae recorded in the Chinese Pharmacopeia (1–3). It is derived from the dried stems of *D. officinale*, traditionally considered to be the best tonic. *D. officinale* is a kind of a rare, endangered, and precious folk medicine and food, mainly distributed in Zhejiang, Anhui, and Yunnan provinces in China (4). *D. officinale* is removed from impurities and some fibrous roots are cut off, twisted into a spiral or spring shape while heating, and dried to obtain “Tiepi Fengdou.” Nowadays, due to the extreme scarcity of wild resources and the increasing demand, Tiepi Fengdou has become one of the most expensive tea ingredients in the world. *Dendrobium* genus has high species diversity, inconspicuous macroscopic identification features, and a high price of Tiepi Fengdou, resulting in adulteration, confusion of varieties, and counterfeiting from time to time. *D. officinale* is valued for its medicinal uses and has a history of thousands of years in traditional Chinese medicine (TCM) (5). Medication history shows that *D. officinale* has the effects of clearing internal heat, nourishing Yin, benefiting the stomach, and generating body fluid (6). *D. officinale* possesses hepatoprotective, anti-tumor, hypoglycemic, gastro-protective, anti-inflammatory, immunomodulatory, and vasodilating effects. Anti-inflammatory is one of the most important biological effects of *D. officinale*, which is an essential causative factor in many diseases such as Alzheimer’s disease (AD), type 2 diabetes, and so on (7, 8).

Recently, many components from *D. officinale*, such as alkaloids, flavonoids, amino acids, and polysaccharides have been reported to have important bioactivities, including anti-inflammatory activity (9–12), especially the reports on *D. officinale* polysaccharides (DOPS). DOPS is the indicator component identified under the item of *D. officinale* in the Chinese Pharmacopeia, and many reports confirmed that DOPS exhibited anti-inflammatory activity (13). Previous research mentioned that glucomannans (GMs) from different plants including *D. officinale* played an important role in their anti-inflammatory properties. Furthermore, the high molecular weight may negatively affect their beneficial effect (14). It has also been noted that for some polysaccharides, molecular degradation modification is required to enhance their activity by reducing their molecular weight to improve their solubility in the water phase, and the quality control for DOPS is very difficult and limited (15). It is suggested that the natural oligosaccharides present in the plant itself have a lower molecular weight and greater solubility, foreshadowing that oligosaccharides may exhibit similar or even greater activity. Moreover, there was a lack of studies for the determination of the anti-inflammatory activity of oligosaccharides from *D. officinale* (DOOS), and also the investigation of anti-inflammatory DOOS was insufficient. Therefore, it is hypothesized to establish a fast and effective method to identify and improve the true and false quality of *D. officinale* by exploring anti-inflammatory DOOS markers.

The quality standard of most TCM is the determination of single or multiple active ingredients or characteristic ingredients that are not related to their efficacy. To establish a quality control and quality traceability system of TCM, a new concept of quality marking (Q-marker) has been proposed in recent years. Q-markers refer to the inherent chemical components of TCM or compounds produced during the preparation process. Their biological activities are closely related to the functional properties of TCM, and their chemical structures have been clarified. Q-markers can be closely linked to the material basis, effectiveness, and quality control of TCM. High performance liquid chromatographic (HPLC) fingerprint is a useful tool to identify the authenticity of TCM, evaluate the quality, and distinguish the components of TCM (16). The spectrum–effect relationship that determines the correlations between fingerprints and biological activity is a scientific method for elucidating the pharmacodynamic basis and establishing a method for controlling the quality of TCM. Determining the spectral–effect relationship between fingerprints and biological activity is a scientific method to clarify the basis of pharmacodynamics and establish quality control methods for TCM. It has been used to explore the biologically active components of TCM, combined with various statistical methods such as regression analysis (RA), hierarchical cluster analysis (HCA), principal component analysis (PCA), orthogonal partial least squares discriminant analysis (OPLS-DA), and gray correlation analysis (GCA). Among them, GCA is a simple and effective method to evaluate the relationship of spectrum effects by judging the correlation grade between factors based on the similarity of the geometry of the change curve of each factor (17, 18), although the China State Food and Drug Administration recommends that all chromatographic fingerprints should be evaluated by similarity analysis (SA).

In this study, the HPLC- charged aerosol detector (CAD) fingerprints combined with the inhibition of nitric oxide (NO) secretion of RAW264.7 cells by different batches of DOOS were evaluated by GCA to determine the spectrum–effect relationships of DOOS active components. Meanwhile, the purified oligosaccharide components were characterized and validated for NO inhibition activity *in vitro*. Due to the singularity of the Chinese Pharmacopeia in the selection of quality control index components of *D. officinale*, the opportunity for adulteration and substandard is made available. As a result, DOOS may potentially become a powerful quality marker for the identification and quality control of *D. officinale*.

## Materials and Methods

### Materials

Forty-seven batches of *D. officinale* raw material samples (S1–S47) were collected from Zhejiang, Fujian, Yunnan, and other regions at diverse periods. The various sources of the samples are shown in Supplementary Table 4 and were authenticated by *Prof*. L.H. Wu from the Institute of Chinese Materia Medica, Shanghai University of Traditional Chinese Medicine, and the reference batch (S48) was purchased from China National Institute for Food and Drug Control.

### Chemicals and Reagents

Deionized water was obtained through a Milli-Q water purification system (Millipore, Bedford, MA, United States). HPLC grade acetonitrile was purchased from Aladdin Chemistry Co., Ltd (Shanghai, China). Formic acid for chromatographic analysis was obtained from J.T. Baker (Phillipsburg, PA, United States). Ammonium formate was provided by Sigma Aldrich (Sigma Aldrich, St. Louis, MO, United States). The standards of sucrose, maltotriose, maltotetraose, maltopentaose, maltohexaose, maltoheptaose, maltooctaose, mannobiose, mannotriose, mannotetraose, mannopentaose, and mannohexaose were purchased from Aladdin (Shanghai, China). Monosaccharides D-glucose (Glc), D-galactose (Gal), L-arabinose (Ara), L-rhamnose (Rha), D-mannose (Man), D-xylose (Xyl), and L-fucose (Fuc), as well as trifluoroacetic acid (TFA) were obtained from Sigma Aldrich (St Louis, MO, United States). Molecular weight 6100 standard was purchased from Shodex Co. (Tokyo, Japan). CNWBOND Carbon-GCB Cartridge (500 mg, 6 mL) was from Shanghai Ampel Scientific Instrument (China) and D_2_O (99.8 Atom% Deuterium) was from Schweres Wasser, United States). Fetal bovine serum (FBS), Dulbecco’s modified Eagle medium (DEME medium), PBS, trypsin, and penicillin/streptomycin were obtained from Gibco (Grand Island, NY, United States). LPS (lipopolysaccharide) was obtained from Sigma Aldrich (St Louis, MO, United States). CCK-8 was purchased from Yeasen Biotech Co., Ltd. (Shanghai, China). NO assay kit was purchased from Nanjing Jiancheng Bioengineering Institute (Nanjing, China). Commercial ELISA kits were bought from Sigma Aldrich (Sigma Aldrich, St. Louis, MO, United States). RAW264.7 cells were obtained from the American Type of Culture Collection (ATCC, Manassas, VA). All chemicals used were of analytical-reagent grade unless stated otherwise.

### High Performance Liquid Chromatographic-Charged Aerosol Detector Fingerprint Assessment

#### High Performance Liquid Chromatographic-Charged Aerosol Detector Sample Preparation

Extraction conditions were designed to maximize DOOS preparation yield. The three-dimensional response surface and two-dimensional contours drawn by Design Expert software showed the optimized extraction parameters of the DOOS as follows: extraction temperature 70°C, extraction time 2 h, extraction times twice, and the ratio of solution to material 60 mL/g by Design Expert software (Supporting information, Part I).

All batches of samples were dried at 60°C using hot air to a constant weight and then ground to a fine powder that could pass through a 50-mesh sieve. Each batch of sample powder was extracted with 0.5 g under the same conditions. The extraction conditions were controlled as follows: extraction temperature 70°C, extraction time 2 h, extraction times twice, and the ratio of solution to material 60 mL/g. The extraction solution was prepared by weight-relief method to compensate for the weight lost during the two extractions. The aqueous extracts were combined and centrifuged at 10,000 × g for 10 min, then 30 mL of supernatant was precisely removed, and 120 mL of anhydrous ethanol was added to the supernatant to ensure that the final concentration of ethanol in the mixture was 80% (v/v) and kept at 4°C for 12 h. After centrifuging at 10,000 × g for 10 min, the supernatant was concentrated to dryness and dissolved with 1 mL of deionized water.

Previous extraction studies have shown that oligosaccharides can be largely retained by graphitized carbon (19). In this context, Carbon–GCB SPE Cartridge (CNW, Shanghai) was used to obtain oligosaccharides from solutions. Prior to use, the packing should be washed with 6 mL deionized water, methanol, and deionized water, respectively. A total of 1 mL of the extract obtained by the above method was added and centrifuged (10,000 × g for 10 min), and the supernatant was applied to the activated Carbon-GCB SPE Cartridge. Salts and monosaccharides were washed off with 6 mL of deionized water, while the oligosaccharides were adsorbed to Carbon-GCB SPE Cartridge, then eluted with 6 mL 50% concentration of methanol in deionized water and dried. The samples were dissolved in 0.5 mL of deionized water and kept at 4°C for HPLC-CAD analysis.

#### The Fingerprint Analysis

The HPLC chromatographic fingerprint analysis was performed by HPLC U3000 with a Corona^®^ CAD (Thermo Scientific, Idstein, Germany). A Prevail Carbohydrate ES column (250 mm × 4.6 mm, 5 μm, Grace) was used to separate the samples at 45°C. The mobile phase was composed of acetonitrile as the phase A and 30 mM ammonium formate with 0.3% formic acid in deionized water as the phase B. The gradient elution program was set as follows: 0-60 min, 20–50% B; 60-80 min, 50% B. The injection volume was 10 μL and the flow rate was set to 1 mL/min. The settings for the CAD were as follows: data collection rate, 20 Hz; filter, 5.0; and evaporation temperature, 35°C. Data acquisition and analysis were performed using Thermo ScientificTM Dionex Chromeleon 7.2 SR4 software.

#### Validation of High Performance Liquid Chromatographic-Charged Aerosol Detector Method

To ensure the reliability of the method, precision, repeatability, and stability of the method were verified in this study. The precision of the analysis was determined by injecting the same sample solution six times in succession. The stability of DOOS solution was verified by analyzing at 0, 2, 4, 8, 16 and 24 h, respectively. The repeatability of the method was assessed by analyzing six independently prepared sample solutions from the same batch of *D. officinale*. Finally, the method was evaluated by RSD. The method was evaluated by determining the RSD of relative retention time (RRT) and the relative peak area (RPA) of compounds to assess the precision, stability, and repeatability.

#### Similarity of High Performance Liquid Chromatographic-Charged Aerosol Detector Analysis

Similarity analysis was analyzed by the software Similarity Evaluation System for Chromatographic Fingerprint of Traditional Chinese Medicine software (Version 2004A). The spectrum of sample S48 was used as the reference spectrum, which was obtained from China National Institute for Food and Drug Control; the fingerprints were automatically matched and established. Subsequently, the median method was used to compare the fingerprints of each batch, and the similarity between the reference fingerprint and each batch chromatogram was calculated.

### Hierarchical Clustering Analysis

Hierarchical clustering analysis (HCA) is a multivariate analysis method that classifies samples into clusters (20). In the present study, HCA of 48 batches DOOS was performed using by SPSS 22 (SPSS Inc., United States) software.

### Identification of Common Peaks

#### Isolation and Purification of DOOS

*Dendrobium officinale* powder (500 g) was extracted with the response surface optimized extraction method and the extracts were mixed and concentrated in a rotary evaporator at 60°C under reduced pressure. The concentrated solution was centrifuged at 4,000 × *g* for 20 min to remove insoluble contaminants. To ensure that the final concentration of ethanol in the mixture is 80% (v/v), anhydrous ethanol was added to the supernatant. The supernatant was kept at room temperature for 12 h and collected after centrifugation at 4,000 × g for 20 min. Then the supernatant was concentrated at 60°C under reduced pressure and then lyophilized to obtain crude oligosaccharides.

The crude oligosaccharides were dissolved sufficiently with distilled water and then centrifuged at 10,000 × g for 10 min. The supernatant was loaded on a graphitized carbon–diatomite column which was mixed in a ratio of 1:1. Then it was eluted with distilled water (DOOS-W), 20% ethanol solution (DOOS-20), and 50% ethanol solution (DOOS-50), respectively. DOOS-20 was further purified using a CAPCELL PAK C18-AQ (10 mm × 250 mm, 5 μm) preparative column at a flow rate of 2 mL/min and collected separately according to the chromatographic results. Each purified portion of DOOS-20 (10 mg/mL) was analyzed by the established HPLC-CAD fingerprint method and compared with the behavior of the standards (10 mg/mL), respectively.

#### Monosaccharide Composition Analysis

DOOS-20 of each purified oligosaccharide was weighed at 1 mg and then hydrolyzed with 2 M TFA for 2 h at 100°C. After neutralization with 2 M NaOH, the oligosaccharides were reduced with NaBH_4_ in a water bath at 40°C for 90 min according to the method of Lehrfeld (21) and acetylated with Ac_2_O for 10 min at 40°C. The resulting alditol acetates were subjected to GC-Mass Spectrum analysis. Data were analyzed with Agilent MassHunter Workstation.

#### High-Performance Liquid Chromatography Quadrupole Time-of-Flight Spectroscopy Analysis

The HPLC-Quadrupole Time of Flight (QTOF)-Mass Spectrum (MS) analysis was performed on an Agilent 1290 Infinity system coupled to an Agilent 6546 Quadrupole Time of Flight mass spectrometer (Santa Clara, CA, United States) equipped with an electrospray ionization (ESI) interface. The Mass Spectrum system was run in negative ionization mode and the Mass Spectrum conditions were as follows: gas temperature 320°C, drying gas flow 8 L/min, nebulizer 35 psi, sheath gas temperature 350°C, sheath gas flow 11 L/min, capillary voltage 3500 V, skimmer voltage 65 V, octupole RF voltage 750 V, fragmentor voltage 175 V. The full-scan mass range was 50–1,700 Da. Accurate mass measurements were obtained by using a calibration solution which contains the internal reference masses at *m/z* 112.9855 and 1033.9881 in negative mode. Data were analyzed with Agilent MassHunter Workstation.

#### Nuclear Magnetic Resonance Analysis

DOOS-20 of each purified oligosaccharide (5–20 mg) was dissolved in 0.4 mL D_2_O (99.8 Atom% Deuterium; Schweres Wasser, United States), separately. Nuclear magnetic resonance (NMR) spectra were recorded using the Bruker Avance III 600 Spectrometer (Bruker Instruments, Inc., Billerica, MA, United States) at 25°C. All the experiments were recorded, and data were processed using standard Bruker software and MestReNova.

### Anti-inflammatory Activity of DOOS *in vitro*

#### Cell Culture and Cytotoxicity

RAW264.7 cells were cultured at 37°C in Dulbecco’s modified eagle’s medium (DMEM) containing 1% PS (Penicillin–Streptomycin) solution and 10% FBS in a 5% CO_2_ atmosphere. RAW267.4 cells were inoculated overnight in 96-well plates at a density of 4 × 10^4^ cells/well and then pretreated with different concentrations of DOOS (40, 20, 10, 5, 2.5 mg/mL, the concentration of the drug is calculated by the mass of the corresponding raw herb) for 1 h, followed by stimulation with 1 μg/mL of LPS for 24 h. Then 20 μL of CCK-8 solution was added to each well. Absorbance was measured at 450 nm using a microplate reader (Biotek, United States) after 30 min incubation. All experiments were performed in triplicate and repeated at least three times. Data are expressed as a percentage relative to untreated control cells.

#### Nitrite Determination

RAW267.4 cells were inoculated overnight in 96-well plates at a density of 4 × 10^4^ cells/well and then pretreated with different concentrations of DOOS (20, 10, 5, 2.5, 1.25, 0.625 mg/mL, the concentration of the drug is calculated by the mass of the corresponding raw herb) for 1 h. In addition, aminoguanidine hydrochloride (50 μg/mL) was used as a positive control (22). After incubation for 1 h, LPS was added at a concentration of 1 μg/mL and incubated for 24 h. The concentration of nitrite accumulated in the culture solution was measured according to the Griess reaction as an indicator of NO production (23). The absorbance was monitored at 540 nm with a microplate reader. All experiments were performed in triplicate and repeated at least three times.

#### Measurements of Cytokines by Enzyme-Linked Immunosorbent Assay

RAW267.4 cells were inoculated overnight in 24-well plates at a density of 2 × 10^5^ cells/well and then pretreated with different concentrations of DOOS (20, 10, 5, 2.5, 1.25, 0.625 mg/mL, the concentration of the drug is calculated by the mass of the corresponding raw herb) for 1 h. In addition, dexamethasone (40 μM) was used as a positive control (24). After 1 h of incubation, LPS was added at a concentration of 1 μg/mL for 24 h. Culture medium was collected for the determination of inflammatory cytokines. The levels of TNF-α, IL-6, and IL-1β were tested using the commercially available ELISA kits according to the manufacturer’s instruction. All experiments were performed in triplicate and repeated at least three times.

#### Nitrite Determination for Different Batches

In this paper, different batches of DOOS were incubated at the same concentration (5 mg/mL, the concentration of the drug is calculated by the mass of the corresponding raw herb) in a model of LPS-induced inflammation in RAW264.7 macrophages, aminoguanidine hydrochloride (50 μg/mL) was used as a positive control, and the nitrite accumulated in culture medium was detected with Griess reagent. The inhibition rate of NO was calculated to evaluate the anti-inflammatory activity of different batches of DOOS.

#### Nitrite Determination for Each Purified Oligosaccharide of DOOS-20

In the present study, each purified oligosaccharide of DOOS-20 was incubated in the LPS-induced inflammation in RAW264.7 macrophages at the same concentration gradients (400, 200, 100, 50, 25 μg/mL) with aminoguanidine hydrochloride (50 μg/mL) as the positive control. The release of nitrite accumulated in the culture medium was detected with Griess reagent.

### Spectrum-Effect Relationships Analysis

In this study, GCA was used to evaluate the spectrum-effect relationships between common peaks in HPLC-CAD fingerprints and anti-inflammatory activity with SPSS 22. Before data analysis, all raw data should be normalized by equation (1) to eliminate the differences caused by different dimensions, where Xmax and Xmin refer to the maximum and minimum values in the sequence, respectively.


(1) $$\text{X}=\frac{\text{X-Xmin}}{\mathrm{Xmax}-\mathrm{Xmin}}$$

The inhibition rate of LPS-induced NO secretion from RAW264.7 cells by different batches of DOOS was used as the reference sequence and recorded as X_0_ (k), and the peak area of the common peaks shared by the fingerprints of each batch of DOOS was used as the comparison sequence and recorded as Xi (k). The correlation coefficient (ξ_*i*_(k)) and the gray correlation grade (γ_*i*_) were calculated as follows:


(2) $$\xi_{i}\,\left(\text{k}\right)=\frac{\min_{\text{i}}{\text{min}}_{\text{k}}\left|X_{0}\,\left(k\right)-X_{i}\,\left(k\right)\right|+\rho \,\max_{i}{\text{max}}_{k}\left|X_{0}\,\left(k\right)-X_{i}\,\left(k\right)\right|}{\left|X_{0}\,\left(k\right)-X_{i}\,\left(k\right)\right|+\rho \,\max_{i}{\text{max}}_{k}\left|X_{0}\,\left(k\right)-X_{i}\,\left(k\right)\right|}$$

(3) $$\gamma_{i}=\frac{1}{N}\,\sum_{k\,1}^{n}\xi_{i}\,\left(\text{k}\right)$$

where k is different batches of DOOS, ρ is the distinctive coefficient lying between 0 < ρ < 1 and it is generally set as 0.5, and max_*i*_ max_*k*_ |*X*_0_(*k*)−*X*_*i*_(*k*)| and min_i_ min_k_ |*X*_0_(*k*)−*X*_*i*_(*k*)| represent the maximum and the minimum difference between the two levels, respectively.

### Data Analysis

The experimental data are expressed as the means ± SD and analyzed with Graphpad Prism 7. One-way ANOVA was used to examine differences among groups. The value of *P* < 0.05 was considered statistically significant.

## Results and Discussion

### Validation of High Performance Liquid Chromatographic-Charged Aerosol Detector Method

The HPLC-CAD method was established for the fingerprinting of DOOS, as well as the precision, stability, and reproducibility of the method were evaluated. According to the results shown in the Supplementary Table 5, peak 1 was used as the reference peak, the RSD values of RRT and RPA for precision, stability, and repeatability did not exceed 3%, indicating that the apparatus precision was satisfactory, the method repeatability was adequate, and the sample solutions were stable within 24 h.

### Fingerprint Analysis and Similarity Evaluation

**FIGURE 1:**
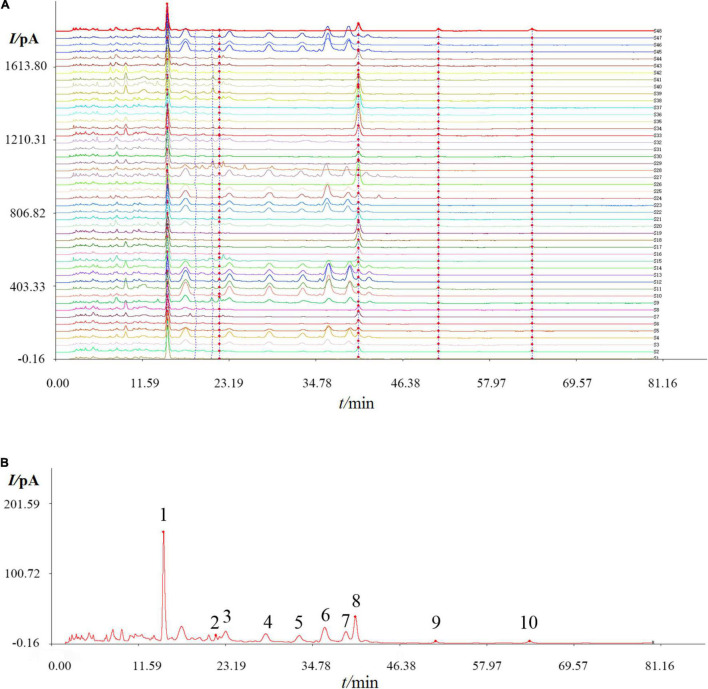
High performance liquid chromatographic (HPLC) fingerprint of 48 batches of DOOS **(A)**, and the reference chromatogram **(B)** that was obtained by using the software Chinese Medicine Fingerprint Similarity Evaluation (2004A Version). Common peaks were marked with 1–10 in the chromatogram.

Graphitized carbon can be used as a solid-phase extraction cartridge to purify oligosaccharides from the solution (25). In this context, Carbon-GCB SPE Cartridge was used to purify the oligosaccharides from the extracts, and different batches of DOOS were analyzed by HPLC-CAD. A total of 48 batches of DOOS samples were analyzed by using the software Chinese Medicine Fingerprint Similarity Evaluation (2004A Version). The HPLC chromatogram was recorded and the results are shown in Figure 1A, and ten common characteristic peaks were observed. The spectrum of standard sample S48 was selected as the reference spectrum, and the software automatically matched the peaks by multi-point calibration. A reference fingerprint was generated by the selected median method, as shown in Figure 1B. The similarity values between the chromatogram for each batch were calculated, and the results are shown in Supplementary Table 6. According to the result listed in Supplementary Table 6, the similarity index was in a range of 0.222–0.995, indicating that there was a large difference between batches of different origins, thus it is reasonable to suspect that it may be due to differences in climate and growth environment of different origins.

### Results of Hierarchical Clustering Analysis

**FIGURE 2:**
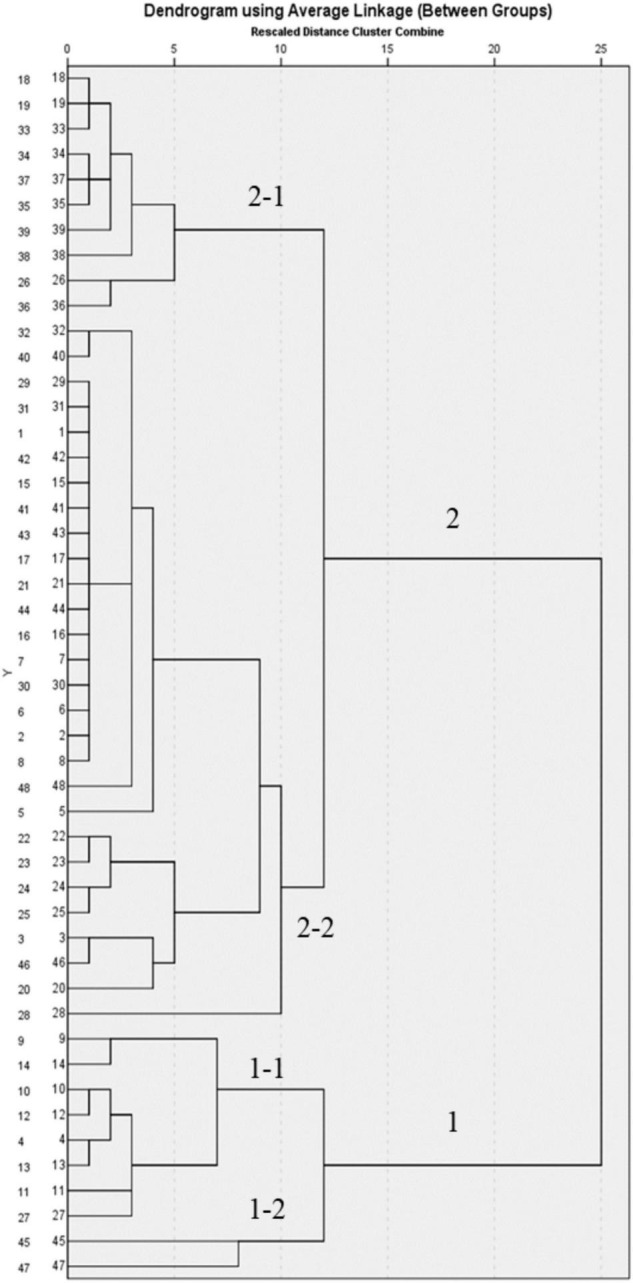
Dendrograms of 48 batches of DOOS resulting from hierarchical clustering analysis.

To validate the results of the SA and further elucidate the similarity relationships among these samples, HCA analysis was performed and the results are shown in Figure 2. HCA in this study showed that the specimens could be divided into two clusters, where S4, S9, S10, S11, S12, S13, S14, S27, S45, and S47 were classified in group 1 and the rest were in group 2, which was divided into two subgroups again. S45 and S47 were in group 1–2 and the others were in group 1–1, while S18, S19, S26, S33, S34, S35, S36, S37, S38, and S39 were in group 2-1 and the others were in group 2-2. Compared with the results of SA, group 1 had higher similarity with the reference crude drug, while group 2 was less similar compared with other batches, suggesting that origins and harvesting season had a greater influence on the quality of *D. officinale*.

### Identification of Common Peaks

#### Isolation and Purification of DOOS

**FIGURE 3:**
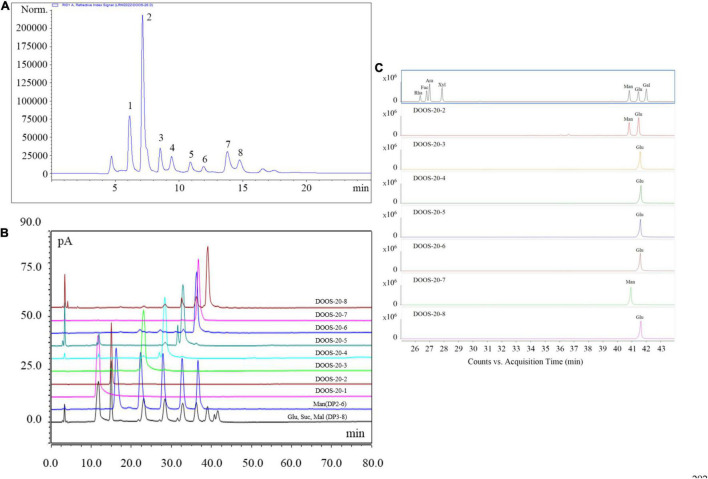
**(A)** CAPCELL PAK C18-AQ preparative column chromatography elution curve of DOOS-20; **(B)** HPLC-CAD chromatography results of DOOS-20 purified sections and mixed standards of glucose, sucrose, malto-oligosaccharide standard (DP3-8), and mannan-oligosaccharide standard (DP2-6) control; **(C)** Monosaccharide composition analysis of DOOS-20 purified sections.

The crude oligosaccharides were extracted from the stems of *D. officinale* by response surface optimization method and fractionated by graphitized carbon-diatomite column chromatography to obtain 20% ethanol solution (DOOS-20), which were further purified by CAPCELL PAK C18-AQ preparative column (Figure 3A). The purified samples were lyophilized and analyzed by HPLC-CAD at a concentration of 10 mg/mL and checked against mixed standards of glucose, sucrose, malto-oligosaccharide (DP3–DP8), and mannan-oligosaccharide (DP2–DP6) mixed standards (Figure 3B). As shown in Figure 3B, DOOS-20-1 was a monosaccharide and would not be considered for subsequent studies of oligosaccharide components. DOOS-20-2, DOOS-20-3, DOOS-20-4, DOOS-20-5, DOOS-20-6, DOOS-20-7, and DOOS-20-8 may be DP2-DP8, respectively. However, peak 9 and peak 10 in Figure 1 cannot be obtained due to low yield rates.

#### Monosaccharide Composition Analysis

The results of monosaccharide composition are shown in Figure 3C. Compared with the monosaccharide standards, the monosaccharide peaks of DOOS-20-3, DOOS-20-4, DOOS-20-5, DOOS-20-6, and DOOS-20-8 were glucose and the monosaccharide peak of DOOS-20-7 was mannose. The monosaccharide composition of DOOS-20-2 was mannose and glucose.

#### High Performance Liquid Chromatographic-Quadrupole Time of Flight-Mass Spectrum Analysis

In the present study, HPLC-Quadrupole Time of Flight-Mass Spectrum was applied in negative ion mode to determine the quasi-molecular ions [M–H] ^–^ of each purified section of DOOS-20. The information was presented in Table 1 and Supplementary Figure 5, including quasi-molecular ions [M–H] ^–^, molecular formula, and degree of polymerization (DP).

**TABLE 1:** Structure identification of each purified section of DOOS-20.

Sample	[M–H]^–^ (*m/z*)	Δ ppm	Molecular Formula	monosaccharide composition	DP	Structure
DOOS-20-2	341.1092	0.59	C_12_H_22_O_11_	Man, Glu*	2	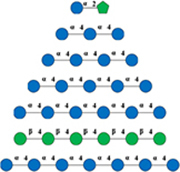
DOOS-20-3	503.1617	−0.17	C_18_H_32_O_16_	Glu	3		
DOOS-20-4	665.2150	0.46	C_24_H_42_O_21_	Glu	4		
DOOS-20-5	827.2676	0.17	C_30_H_52_O_26_	Glu	5		
DOOS-20-6	989.3204	0.09	C_36_H_62_O_31_	Glu	6		
DOOS-20-7	989.3204	0.09	C_36_H_62_O_31_	Man	6		
DOOS-20-8	1151.3730	−0.08	C_42_H_72_O_36_	Glu	7		

*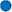 : Glucose; 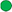 : Mannose; 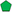 : Fructose. DP: Degree of Polymerization.*

*The reduction of fructose produces a mixture of sorbitol and mannitol, resulting in the appearance of a glucose peak together with a mannose peak.

#### Nuclear Magnetic Resonance Analysis

To further confirm the oligosaccharide structure of each purified section of DOOS-20, Nuclear magnetic resonance analysis was performed. According to the characteristic signals, the ^1^H, ^13^C, and DEPT 135 Nuclear magnetic resonance spectra are shown in Supplementary Figure 6. Integrating the monosaccharide composition, molecular weight, and Nuclear magnetic resonance spectra, the following is known: DOOS-20-2 (peak 1) is sucrose (26). In the monosaccharide composition analysis, the reduction of fructose produces a mixture of sorbitol and mannitol, resulting in the appearance of a glucose peak together with a mannose peak. In accordance with the Nuclear magnetic resonance spectra as well as the monosaccharide composition, it is known that DOOS-20-3 (peak 3), DOOS-20-4 (peak 4), DOOS-20-5 (peak 5), DOOS-20-6 (peak 6), and DOOS-20-8 (peak 8) were maltotriose, maltotetraose, maltopentaose, maltohexaose, and maltoheptaose (27), respectively. DOOS-20-7 (peak 7) was mannohexaose (28).

### Anti-inflammatory Activity of DOOS *in vitro*

#### Effect of DOOS on Cell Viability

**FIGURE 4:**
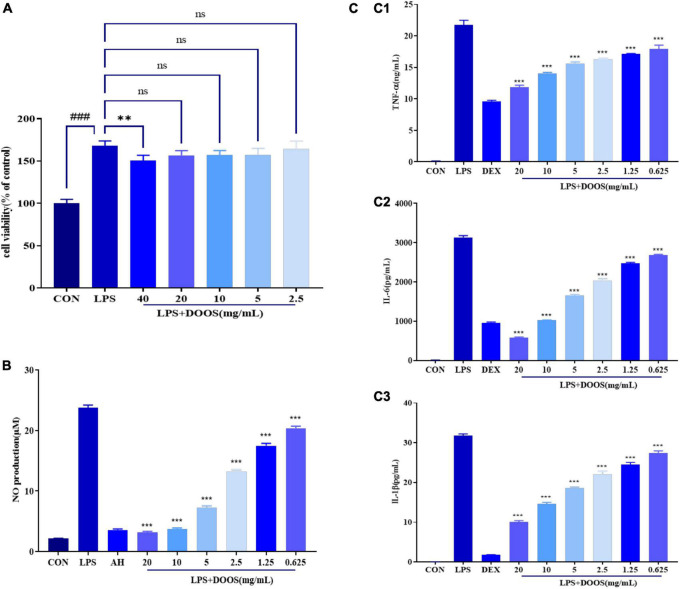
**(A)** Effect of DOOS on the viability of RAW 264.7 cells. RAW 264.7 cells were incubated with different concentrations of DOOS and LPS (1 μg/mL) for 24 h. Cell viability was determined by CCK-8 assay. **(B)** Effects of DOOS on the production of NO in LPS-induced RAW 264.7 cells. The cells were treated with different concentrations of DOOS for 1 h prior to stimulation and LPS (1 μg/mL) for 24 h. **(C)** Effects of DOOS on TNF-α **(C1)**, IL-6 **(C2)**, and IL-1β **(C3)** secretion. The cells were treated with different concentrations of DOOS for 1 h prior to stimulation and LPS (1 μg/mL) for 24 h. Then the supernatant was collected to test cytokines production by ELISA. Values show the means and standard deviations of different concentrations performed in triplicate. ***p < 0.001 and **p < 0.01 vs. LPS group; ns, no significance; ^###^*p* < 0.001 vs. control group.

Before assessing the potential anti-inflammatory activity of DOOS, the effect of DOOS on RAW 264.7 cell viability was first assayed and the results are shown in Figure 4A. The results of the CCK-8 assay showed that DOOS at concentrations of 40, 20, 10, 5, 2.5 mg/mL (concentration of the drug is calculated by the mass of the corresponding raw herb) had no cytotoxic effect on RAW 264.7 cells. It was confirmed that the effect of DOOS on RAW 264.7 cells was not caused by the reduction of cell viability.

#### Nitrite Determination

Macrophage cells play an essential role in the inflammatory process, and lipopolysaccharide (LPS) is one of the most widely used pro-inflammatory stimulators that can activate macrophages and trigger the inflammatory response (29). Recognition and binding of LPS by Toll-like receptor 4 (TLR4) or CD14 specific receptors on the cell membrane leads to the activation of macrophages and increased secretion of inflammatory mediators such as NO (30). The accumulation of the stable metabolite nitrite in the culture supernatant was measured with Griess reagent to evaluate the effect of DOOS on NO production by RAW 264.7 cells. According to the results shown in Figure 4B, compared with the control group, LPS significantly (*p* < 0.001) promoted NO production in macrophages. Furthermore, compared with the group that was treated with LPS, pretreatment with DOOS resulted in an inhibition of the NO production in RAW 264.7 cells, and a clear concentration-dependent manner has been shown between different concentrations, which indicated that DOOS had a strong anti-inflammatory activity.

#### Measurements of Cytokines by Enzyme-Linked Immunosorbent Assay

Macrophages play an essential role in the inflammatory response through the release of a variety of factors, and in addition to NO, inflammatory cytokines such as TNF-α, IL-6, and IL-1β were used as representative cytokines in response to an activating stimulus (e.g., LPS) (31, 32). The concentrations of TNF-α, IL-6, and IL-1β in the culture medium were measured using commercial ELISA kits and the results are shown in Figure 4C. In comparison with the control group, LPS significantly (*p* < 0.001) increased the release of TNF-α (C1), IL-6 (C2), and IL-1β (C3). Furthermore, incubation with different concentrations of DOOS could effectively reduce the release of inflammatory cytokines and had a significant concentration-dependent manner, suggesting that DOOS had an inhibitory effect on the release of inflammatory cytokines.

#### Nitrite Determination for Different Batches

**FIGURE 5:**
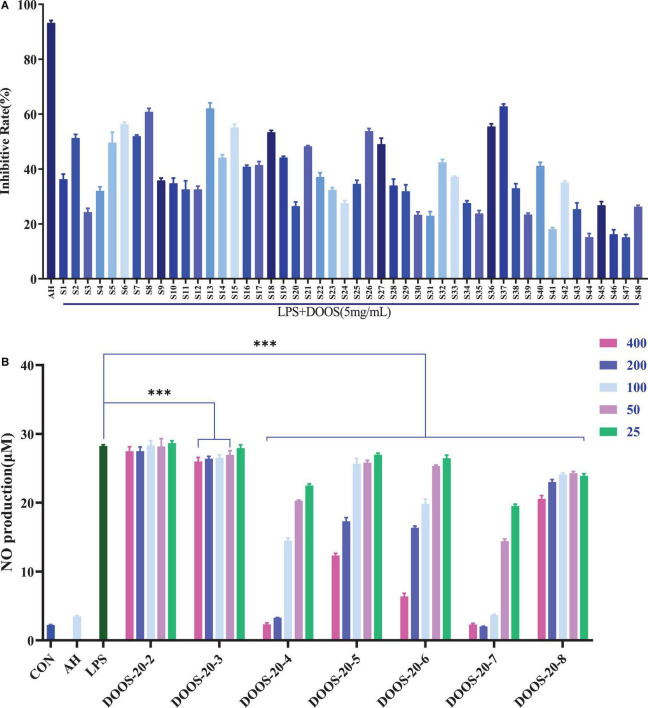
**(A)** Nitric oxide (NO) inhibition rate of LPS induced RAW 264.7 cells with different batches of DOOS. The cells were treated with different batches of DOOS (5 mg/mL, the concentration of the drug is calculated by the mass of the corresponding raw herb) for 1 h prior to stimulation and LPS (1 μg/mL) for 24 h. Values show the means and standard deviations of different concentrations performed in triplicate. **(B)** Effects of DOOS-20 at different concentration gradients (400, 200, 100, 50, 25 μg/mL) in each purified section on production of NO in LPS induced RAW 264.7 cells. The cells were treated with different concentration sof DOOS-20 each purified section for 1 h prior to stimulation and LPS (1 μg/mL) for 24 h. Values show the means and standard deviations of different concentrations performed in triplicate. ***p < 0.001 significantly different from the LPS group; ns, no significance.

The DOOS could differentially inhibit the secretion of NO and inflammatory factors such as TNF-α, IL-6, and IL-1β, and it has a clear concentration-dependent manner between different concentrations, especially, the potential for inhibiting NO secretion is remarkable. Therefore, NO inhibition rate was selected to evaluate the anti-inflammatory activity of different batches of DOOS in this study. According to the results shown in Figure 5A, it indicated that different batches of DOOS exhibited a significant difference in the rate of inhibition of LPS-induced NO secretion from RAW 264.7 macrophages under the same dose conditions, in which S8 (Wuyi, Zhejiang), S13 (Yandang Mountain, Zhejiang), and S37 (Gengma, Yunnan) showed the strongest inhibition of NO secretion. It suggested that the differences in the NO secretion inhibitory activity of DOOS from different batches may be related to the differences in the content of anti-inflammatory components in different batches.

### Established and Verified Spectrum–Effect Relationships Based on Oligosaccharides

#### Established Spectrum–Effect Relationships Between High Performance Liquid Chromatographic -Charged Aerosol Detector Fingerprints and Anti-inflammatory Activities

**FIGURE 6:**
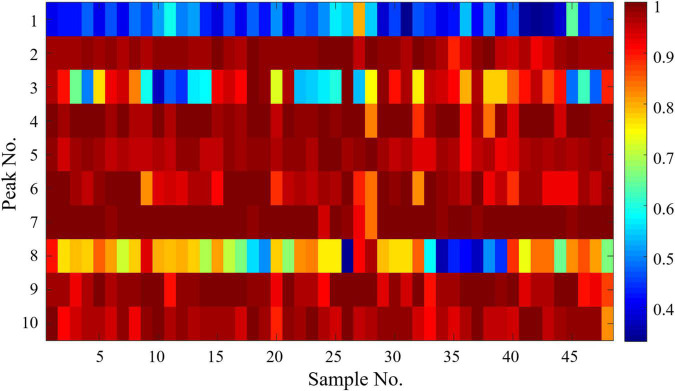
Spectrum-effect relationships between HPLC-CAD fingerprints and anti-inflammatory activities.

Gray correlation analysis was performed with SPSS 22 software to evaluate the spectrum–effect relationships between the 10 common peak areas in the HPLC-CAD fingerprints and anti-inflammatory activities, and the results of the correlation coefficients were expressed as a heat map shown in Figure 6. The gray correlation grade was calculated and ranked according to the gray correlation coefficient, which was shown in Table 2. Based on correlation coefficients, the contribution of each peak to the anti-inflammatory activity was in the order of P7 > P4 > P2 > P9 > P5 > P10 > P6 > P3 > P8 > P1, where the correlation coefficient of peak 1 was below 0.5, indicating that peak 1 had little correlation with the anti-inflammatory activity. Except for peak 3 and peak 8, the correlation coefficient of the rest chromatographic peaks was above 0.9, indicating that these peaks (P7, P4, P2, P9, P5, P10, P6) correlate well with anti-inflammatory activity, and could as the quality makers of DOOS. Based on the results of inhibition of LPS-induced NO secretion by RAW264.7 macrophages *in vitro* in Figure 5A, GCA can be used to assess the correlation between the efficacy index and the chromatographic peaks and provide a possible prediction of the active components.

**TABLE 2:** Gray correlation grade for the common peak areas and their order.

Peak No.		Correlation Coefficient	Order
P1	Monosaccharide	0.460 ± 0.082	10
P2	Sucrose	0.974 ± 0.020	3
P3	Maltotriose	0.773 ± 0.189	8
P4	Maltotetraose	0.975 ± 0.038	2
P5	Maltopentaose	0.966 ± 0.019	5
P6	Maltohexaose	0.955 ± 0.046	7
P7	Mannohexaose	0.988 ± 0.025	1
P8	Maltoheptaose	0.720 ± 0.160	9
P9	–	0.969 ± 0.033	4
P10	–	0.962 ± 0.032	6

#### Verified the Spectrum-Effect Relationships Based on Purified Oligosaccharides

To examine the anti-inflammatory activity of each purified oligosaccharide (P2–P8) from DOOS-20 at different concentration gradients (400, 200, 100, 50, 25 μg/mL), the secretion of NO was measured with Griess reagent. As shown in Figure 5B, the differences between different concentration gradients of P-2 (sucrose) on the secretion of NO from RAW264.7 macrophages compared with the LPS group were not statistically significant. The rest of DOOS-20 purification sections had a certain inhibitory effect on NO secretion from macrophages, among which P-4 (maltotetraose), P-5 (maltopentaose), P-6 (maltohexaose), and P-7 (mannohexaose) had a more significant NO inhibitory effect. It indicated that P-4 (maltotetraose), P-5 (maltopentaose), P-6 (maltohexaose), and P-7 (mannohexaose) could be the quality markers for quality control of *D. officinale*.

## Discussion

*Dendrobium officinale*, Chinese name Tiepi Shihu, is derived from the dried stems of *Dendrobium Kimura*, traditionally considered to be the best tonic for nourishing the stomach, nourishing body fluid, and strengthening the immunity (33). Polysaccharide is the indicator component identified under the item of *D. officinale* in the Chinese Pharmacopeia (Part I). However, only the total sugar content and monosaccharide composition ratio of polysaccharide from *D. officinale* are generally limited, which provides an opportunity for the existence of *D. officinale* fake products. Therefore, it is urgent to establish a fast and effective method to identify and improve the true and false quality of *D. officinale*.

Carbohydrates are the most abundant natural products and can be classified according to their DP, initially divided into three main groups, namely monosaccharides (DP 1), oligosaccharides (DP 2–10), and polysaccharides (DP > 10). Functional oligosaccharides are indigestible and insensitive to human digestive enzymes, resulting in various pharmacological effects, for example, anti-inflammatory effect (34). *D. officinale* has the effects of clearing internal heat, nourishing Yin, benefiting the stomach and generating body fluid. Polysaccharides of *D. officinale* has good anti-inflammatory effects, consistent with TCM efficacy (13). GM is the main component of *Dendrobium* polysaccharide that exerts anti-inflammatory effect and higher molecular weight may have a negative impact on the anti-inflammatory effect (14), while compared with polysaccharide, oligosaccharide has lower molecular weight and better water solubility and are easily absorbed through the intestine (35). In our study, the HPLC-CAD fingerprints combined with the inhibition of NO secretion of RAW264.7 cells by different batches of DOOS were evaluated by GCA to determine the spectrum-effect relationships of DOOS active components. Meanwhile, the purified oligosaccharide components were characterized and validated for NO inhibition activity *in vitro*, and the results indicated that P-4 (maltotetraose), P-5 (maltopentaose), P-6 (maltohexaose), and P-7 (mannohexaose) could be the quality markers for quality control of *D. officinale*. As far as we know this is the first time that the anti-inflammatory effects of maltotetraose, maltopentaose, maltohexaose, and mannohexaose in *D. officinale* were reported.

In 1990, Alpert first proposed the separation mode of hydrophilic interaction liquid chromatography (HILIC) (36), which solved the problem that compounds with high polarity could not be well retained in reversed-phase liquid chromatography (RP-LC) (37), and HILIC was widely used for the analysis of oligosaccharides (38, 39). HPLC with CAD, refractive index detection (RID), or evaporative light-scattering detection (ELSD) is often used for oligosaccharide analysis. RID, a traditional detector, has good linearity and stability and has a strong quantitative ability, but CAD detector could obtain a better quantitative limit, sensitive, reproducibility, and linearity than RID. HPLC with CAD is a new detection method that is developed on the basis of ELSD. Similar to ELSD, the response of CAD does not depend on the strong chromophore of the analyte structure, and CAD provides higher sensitivity in comparison to ELSD, making it a powerful tool for the determination of oligosaccharides (40–42). In our study, the fingerprints of DOOS were established by HPLC with HILIC model and CAD detector. Chromatographic fingerprinting is an effective method to evaluate the quality of herbal medicines, but it is difficult to reflect the active ingredient groups in herbal medicines (16). Therefore, it is imperative to establish spectrum–effect relationships between DOOS fingerprints and anti-inflammatory activity. GCA is an essential method of gray system theory, which is a simple and effective method to evaluate the relationship of spectrum-effect by judging the correlation grade between factors based on the similarity of the geometry of the change curve of each factor (17, 18). Many reports about the spectrum–effect relationship study by GCA, for example, reverse tracing anti-thrombotic active ingredients from dried *Rehmannia Radix* was studied based on multidimensional spectrum–effects relationship analysis of steaming and drying for nine cycles (43). Spectrum–effect relationship between constituents absorbed into blood and bioactivities of Baizhu Shaoyao San before and after processing on ulcerative colitis rats by GCA method (44). The spectrum–effect relationships between chemical fingerprints, and the analgesic and anti-inflammatory effects of *Rubia cordifolia L.* extract were established by the GCA method (20). Four chemometrics named PCA, GCA, partial least squares regression (PLSR), and the bivariate correlations analysis (BCA) were applied to construct spectrum–effect relationship between the UPLC fingerprints and biological activities of *Rosa rugosa*. The spectrum–effect relationship study revealed that di-O-galloyl-HHDP-glucoside, galloyl-HHDP-glucoside, and avicularin were more relevant to the antidiabetic activity. Di-O-galloyl-HHDP-glucoside, galloyl-HHDP-glucoside, and ellagic acid were the main antioxidant components of *R. rugosa* (45). In this study, the GCA method was used to evaluate the spectrum–effect relationships between the 10 common peak areas in the HPLC-CAD fingerprints and anti-inflammatory activities, and purified oligosaccharides were used to confirm the GCA results, and the results indicated that maltotetraose, maltopentaose, maltohexaose, and mannohexaose were relevant to anti-inflammatory effects and could be as the quality markers for quality control of *D. officinale*.

Polysaccharide, as one of the main bioactive components in *D. officinale*, was mainly composed of glucose and mannose (Manp: Glcp = 2.01:1.00–8.82:1.00), along with galactose, xylose, arabinose, and rhamnose in different molar ratios and types of glycosidic bonds. The basic skeleton of purified *D. officinale* polysaccharides has been elucidated. The backbone skeleton of *D. officinale* polysaccharides consists of 1,4-β-D-Man*p*, 1,4-β-D-Glc*p*, 1,3,6-β-D-Man*p*, 1,3-β-D- Man*p*, 1,2-β-D-Glc*p*, 1,4-α-D-Man*p*, and 1,6-α-D-Glc*p* (46). An oligosaccharide-marker approach was developed for quality assessment of polysaccharides in *D. officinale* by UPLC-Q-TOF/Mass Spectrum spectrometry previously. The method involved partial acid hydrolysis of *D. officinale* polysaccharide (DOPS) followed by p-aminobenzoic ethyl ester (ABEE) derivatization. Two ABEE-labeled oligosaccharides namely, Te-Man-ABEE and Pen-Man-ABEE, were selected as chemical markers due to their high specificity in herb formula. The linear relationship between the content of these two markers and the content of DOPS was then successfully established, respectively. The linear relationship was further transformed to that between the peak area of chemical markers and DOPS content so that chemical makers were not necessary to be isolated for analysis (47). The original oligosaccharides (no ABBE derivatization) were mannotetraose and mannopentaose, respectively. The structures of mannotetraose and mannoproteins are 1,4-β-D-Man*p*. It is obvious that the partial acid hydrolysis of polysaccharide and ABEE derivatization have the limitation of structural modification and complex procedures. In our study, the proposed Q-markers for quality control of *D. officinale* are maltotetraose, maltopentaose, maltohexaose, and mannohexaose, and among them maltotetraose, maltopentaose, maltohexaose have 1,4-α-D-Glc*p*, and mannohexaose has 1,4-β-D-Man*p*; the four oligosaccharides kept the original structure from *D. officinale*. It suggests that the oligosaccharide-marker approach is a simple, stable, and a reliable method for the quality control of herb medicines or nutritious foods.

## Conclusion

In the present study, HPLC fingerprints were firstly combined with anti-inflammatory activity to evaluate DOOS spectrum–effect relationship by GCA, which provides the possibility to evaluate potential active ingredients and explore the quality markers. The HPLC fingerprints of 48 batches of DOOS were developed and analyzed with SA, and the results showed that the similarity index was in a range of 0.222–0.995, indicating that there was a large difference between batches of different origins. *In vitro* screening experiment indicated that DOOS potentially inhibited NO production and effectively reduced the release of inflammatory cytokines such as TNF-α, IL-6, and IL-1β in RAW 264.7 cells, thereby reducing the inflammatory response of cells. Furthermore, the anti-inflammatory activity of different batches of DOOS was evaluated by NO inhibition as an indicator, and the spectrum–effect relationships were analyzed with HPLC fingerprints by GCA. The oligosaccharides isolated and purified from *D. officinale* were analyzed by monosaccharide composition, HPLC-Quadrupole Time of Flight-Mass Spectrum, and Nuclear magnetic resonance analysis to identify the common peaks as follows: DOOS-20-2 (peak 2) is sucrose, DOOS-20-3 (peak 3), DOOS-20-4 (peak 4), DOOS-20-5 (peak 5), DOOS-20-6 (peak 6), and DOOS-20-8 (peak 8) were maltotriose, maltotetraose, maltopentaose, maltohexaose, and maltoheptaose, respectively, and DOOS-20-7 (peak 7) was mannohexaose. Results of the inhibition of LPS-induced NO secretion by RAW264.7 cells *in vitro* showed that sucrose was not statistically significant compared with the LPS group, and on the other hand, maltotetraose, maltopentaose, maltohexaose, and mannohexaose had a more significant NO inhibitory effect, which was consistent with the correlation coefficient predicted by GCA. Our results showed four DOOS (maltotetraose, maltopentaose, maltohexaose, and mannohexaose) were relevant to anti-inflammatory effects, and could be the quality markers for quality control of *D. officinale*. In conclusion, the strategy of combining chemical fingerprints with pharmacodynamic analysis can provide useful references for the quality evaluation of herbal medicines or nutritious foods.

## Data Availability Statement

The original contributions presented in the study are included in the article/Supplementary Material, further inquiries can be directed to the corresponding authors.

## Author Contributions

RL: methodology, software, validation, formal analysis, investigation, and writing – original draft. SS: methodology, validation, analysis, and interpretation of data. SX: methodology, investigation, and resources. JS: investigation and conceptualization. XG and JW: investigation and resources. HW and SW: funding acquisition, conceptualization, supervision, and writing – review and editing. All authors contributed to the article and approved the submitted version.

## Conflict of Interest

XG was employed by Amway (China) Co., Ltd. The remaining authors declare that the research was conducted in the absence of any commercial or financial relationships that could be construed as a potential conflict of interest.

## Publisher’s Note

All claims expressed in this article are solely those of the authors and do not necessarily represent those of their affiliated organizations, or those of the publisher, the editors and the reviewers. Any product that may be evaluated in this article, or claim that may be made by its manufacturer, is not guaranteed or endorsed by the publisher.
