# Supplementary material for: Quality Markers of Dendrobium officinale by “Oligosaccharide-Spectrum-Effect” Relationships
Source: Front Nutr. 2022 Jun 6;9:914380. doi: 10.3389/fnut.2022.914380 (PMC9221367; doi:10.3389/fnut.2022.914380)
Supplement: Supplementary file 1 [file Data_Sheet_1.docx]

Supporting information

Quality markers of *Dendrobium officinale* by “oligosaccharide-spectrum-effect” relationships

Ruimin Liu^1^, Songshan Shi^1^, Si Xiong^1^, Juan Su^1^, Xiaona Gan^2^, Jianjun Wu^1^, Huijun Wang ^1,^ *, Shunchun Wang ^1,^ *

^1^ The MOE Key Laboratory for Standardization of Chinese Medicines and the SATCM Key Laboratory for New Resources and Quality Evaluation of Chinese Medicines, Institute of Chinese Materia Medica, Shanghai University of Traditional Chinese Medicine, 1200 Cailun Road, Shanghai 201203, PR China

^2^ Nutrilite Health Institute, Amway (China) R&D Center, Shanghai, China, 720 Cailun Road, Shanghai 201203, China.

* Correspondences:

Huijun Wang, Institute of Chinese Materia Medica, Shanghai University of Traditional Chinese Medicine, 1200 Cailun Road, Shanghai 201203, P. R. China; Tel.: +86-21-5132-2511; Fax: +86-21-5132-2519; E-Mail: huijun.wang@outlook.com, ORCID: 0000-0001-6319-498X (H.W.)

Shunchun Wang, Institute of Chinese Materia Medica, Shanghai University of Traditional Chinese Medicine, 1200 Cailun Road, Shanghai 201203, P. R. China; Tel.: +86-21-5132-2511; Fax: +86-21-5132-2519; E-Mail: shunchunwang@126.com, ORCID: 0000-0003-0384-1350 (S.W.)

Part S1 Design of extraction conditions - maximization of the oligosaccharides from *Dendrobium officinale* (DOOS) preparation yield

Introduction

Many extraction methods have been used to extract *D. officinale* polysaccharides (DOPS) and the effects of different extraction methods on the extraction yield, chemical structure, and antioxidant activity of polysaccharides under the same conditions were compared (1). However, DOOS, from the supernatant of water extraction and ethanol precipitation of *D. officinale* stem, is often discarded as redundant residues after DOPS extraction. Moreover, no optimal extraction method for DOOS has been reported so far. In our pre-experiment, the yield of DOOS is not ideal. Therefore, a novel and efficient extraction method is needed. Response surface methodology (RSM) is a mathematical statistical model for improving and optimizing multi-factor processes and determining how individual factors affect the response (2). The major advantage of RSM is that it reduces the number of experiments required to evaluate individual parameters and their interactions (3). Box–Behnken design (BBD) is a type of RSM that requires fewer experiments. Compared to other methods, BBD is more effective and simpler to organize (4). The extraction process of DOOS was optimized using BBD response surface design on the basis of single-factor experiments (5). The main objectives of this section were to optimize conditions of extraction temperature, ratio of solution to material and extraction time for the extraction of oligosaccharides from *D. officinale* stems by RSM coupled with BBD.

Materials and Methods

Materials and reagents

Forty-eight batches of *D. officinale* raw material samples as well as control herbs were collected from Zhejiang, Fujian, Yunnan and other regions at diverse periods. Deionized water was obtained through a Milli-Q water purification system (Millipore, Bedford, MA, USA). HPLC grade acetonitrile was purchased from Aladdin Chemistry Co., Ltd (Shanghai, China). Constant temperature water bath (HWS-24, Shanghai Chengxian instrument technology Co., Shanghai, China) was used for the traditional hot water extraction procedure. Hot air oven (DHG-9053A) was obtained from Yi-heng technology Co., Ltd, Shanghai, China.

Preparation of sample solution and Chromatographic conditions

HPLC-RID sample preparation and quantitation of DOOS

The stems of *D. officinale* were dried in a hot air oven at 60℃ until the weight was constant. Then it was crushed into fine powder by mechanical grinder and passed through 50-mesh sieve to obtain the powder. Precisely weighed dry powder (0.500 g) was extracted with deionized water under the specified extraction conditions. The extraction conditions were controlled by varies of extraction temperature, extraction time, and ratio of solvent to material. The extracts were mixed, 100 mL of deionized water was added and centrifuged at 10000 × g for 10 min. Precision removal of supernatant 10 mL, add 40 mL of anhydrous ethanol to the supernatant to ensure that the final concentration of ethanol in the mixture is 80% (v/v) and keep it at 4℃ for 12h. Centrifuged (10000 × g for 10 min) and precision removal of supernatant 10 mL, drying with nitrogen, then reconstitute with 0.2 mL of deionized water to obtain the DOOS. Each batch of DOOS was prepared in the same way.

Measurements were carried out using Agilent 1100 HPLC with refractive index detector (Agilent, Santa Clara, CA, USA). The column of KS-802 (ID = 8 mm, length = 300 mm; Shodex Co.) was used at 40℃ for sample separation. The injection volume was 10 µL. The sample was eluted with deionized water under a flow rate of 0.80 mL/min. In this part, D-Glucose, sucrose, maltotriose, maltopentaose, and polysaccharide standard (molecular weight of 6100 Da) were used to establish the relationship between molecular weight and elution volume. According to the peak area -concentration standard curve of sucrose standard, the formula for calculating the yield of DOOS was obtained.

Validation of HPLC-RID method

Calibration curves, limits of detection, and quantification

Based on the subsequent analysis of the oligosaccharide composition of DOOS, sucrose standards were selected to establish standard curves for the quantification of oligosaccharide yields. Sucrose standard was weighed accurately and dissolved in deionized water as stock solution. Serial dilutions of the stock solution were made to seven suitable concentrations and a calibration curve was established. Based on the peak response with a signal-to-noise ratio of 3 and 10, the limit of detection (LOD) and limit of quantification (LOQ) were calculated.

Precision, Repeatability, Stability, and Accuracy

To determine the precision of the HPLC-RID method, the repeatability of intra-day and inter-day measurements at low, medium, and high levels was assessed. The samples were analyzed six times in one day for each of the three concentrations separately, thus determining the intra-day precision. Samples of three concentrations were analyzed three times each for three consecutive days to examine the inter-day precision. The repeatability was confirmed by analyzing six parallel samples. The same sample was analyzed six times at different periods (0, 2, 4, 8, 16 and 24 h) to evaluate the stability of the method. The results are expressed as relative standard deviations (RSD).

To evaluate the accuracy of the method, the recovery test was performed by the addition of sucrose of three different masses (0.127, 0.159 and 0.191 mg). Three replicates were performed for the test, and the mean recovery of each sample was calculated. The accuracy of the HPLC method was calculated by the following formula:

$\mathrm{Recovery}\left( \% \right)=\frac{detected amount-original amount}{added amount}\times100\%$ (1)

Design of extraction conditions

Determination of extraction times

Extraction times were set at 1, 2 and 3 times, respectively, while other conditions were setting as follows: ratio of solution to material 30 mL/g, extraction temperature 70℃ and extraction time 1.5 h. Each group is in triplicate. The content of the DOOS yield was calculated.

Single-factor experiments

Single factor experiments were conducted to determine the level of DOOS extraction by RSM as follows: extraction temperature (range from 50 to 100℃), ratio of solution to material (range from 10 to 60 mL/g) and extraction time (range from 0.5 to 3 h). Keeping only one factor constant, extract 0.500 g dried powder to obtain DOOS according to the method above, for each experiment, each group in triplicate.

Optimization of extraction conditions by BBD

The independent variables and their ranges of values were selected on the basis of a preliminary single tests. With the use of Box-Behnken design (BBD), the extraction conditions of DOOS were optimized with three independent variables (X_1_, extraction temperature; X_2_, extraction time; X_3_, ratio of solution to material) at three levels. The yield of oligosaccharide extraction was used as the dependent variable. The responses obtained from each group of experimental designs were evaluated by Design Expert software (Trial Version 8.0.5b, USA). Experimental data was fitted with the following quadratic polynomial model to obtain the regression coefficients:

$Y=b_{0}+\sum_{i=1}^{3} b_{i}x_{i}+\sum_{i=1}^{3} b_{ii}x_{i}^{2}+\sum_{i=1}^{2} \sum_{j=i+1}^{3} b_{ij}x_{i}x_{j}$ (2)

Where Y denotes the response value, b_0_, b_i_, b_ii_ and b_ij_ are the coefficients of the intercept, linear, quadratic and interaction terms, respectively; x_i_, x_i_^2^ and x_i_x_j_ represent linear, quadratic, and interactive terms of coded independent variables, respectively. ANOVA was performed to determine the fitness of the prediction model, and statistical significance was tested with a probability P value of 0.01 or 0.05.

Results

Validation of HPLC-RID method

Phenol-sulfuric acid method is the simplest and most reliable method for carbohydrate analysis (6). However, due to the interference of free monosaccharides in the supernatant affected the accuracy of the method to determine the oligosaccharide content, this method is not suitable for the determination of oligosaccharide content in this experiment. Therefore, a molecular exclusion high performance liquid chromatography was developed in this experiment.

Calibration curves, limits of detection, and quantification

The regression equation of sucrose (Table S1) was linear with the correlation coefficients (R^2^) 0.9999. The LOD and LOQ were 8.23 µg/mL and 18.89 µg/mL, respectively.

Precision, Repeatability, Stability, and Accuracy

The results of the precision experiment were shown in Table S1. The RSD values of intra-day and inter-day precision of the peak areas were less than 3%, indicating that the method is stable. The RSD of repeatability was 0.49%, lower than 3%. The RSD value of the stability was 1.09%, less than 3%, indicating that the sample solution was stable within 24 h. Recovery experimental results (**Table S1**) showed that the overall average recoveries ranged from 100.12% to 104.79%, indicating that the method is suitable for the measurement of DOOS.

Table S1∣Method validation data: linearity, precision, accuracy, LOD and LOQ of sucrose.

| Linearity | |  | | |  |  | |  |
| --- | --- | --- | --- | --- | --- | --- | --- | --- |
| Regression equation | | Linear range (mg/mL) | | | R^2^ | LOD (µg/mL) | | LOQ (µg/mL) |
| y = 119394 x + 2168.4 | | 0.026 – 3.362 | | | 0.9999 | 8.23 | | 18.89 |
|  |  | |  |  | | |  |  |
| Precision | |  | | |  |  | |  |
| Concentration (mg/mL) | | Intra-day (RSD, %, n=6) | | | | Inter-day (RSD, %, n=3) | | |
| 0.026 | | 0.09 | | | | 0.13 | | |
| 0.840 | | 0.41 | | | | 0.49 | | |
| 3.362 | | 1.02 | | | | 1.14 | | |
|  | |  | | |  |  | |  |
| Recovery | |  | | |  |  | |  |
| Add (mg) | | Found (mg) | | | Recovery (%) | | | RSD (%, n = 3) |
| 0.127 | | 0.291 | | | 104.09 | | | 0.7 |
|  |  | 0.29 | | | 103.41 | | |  |
|  |  | 0.289 | | | 102.69 | | |  |
| 0.159 | | 0.318 | | | 100.25 | | | 0.11 |
|  |  | 0.318 | | | 100.12 | | |  |
|  |  | 0.319 | | | 100.35 | | |  |
| 0.191 | | 0.352 | | | 100.91 | | | 0.12 |
|  |  | 0.352 | | | 101.03 | | |  |
|  |  | 0.352 | | | 100.79 | | |  |

Content of oligosaccharides yield determination

To determine the range of time to peak oligosaccharide, D-Glucose, sucrose, maltotriose, maltopentaose, and polysaccharide standard (molecular weight of 6100 Da) were used to establish the relationship between molecular weight and elution volume **(Figure S1**). Samples were injected into HPGPC for analysis, and measurements were analyzed by the GPC software. The content of DOOS was equal to the sum of the peak area between 8.10-10.40 min. The DOOS yield was calculated as follows:

Yield (%) = (A-2168.4)/ 4775760 ×100% (6)

Which A is the sum of the peak area between 8.10-10.40 min.


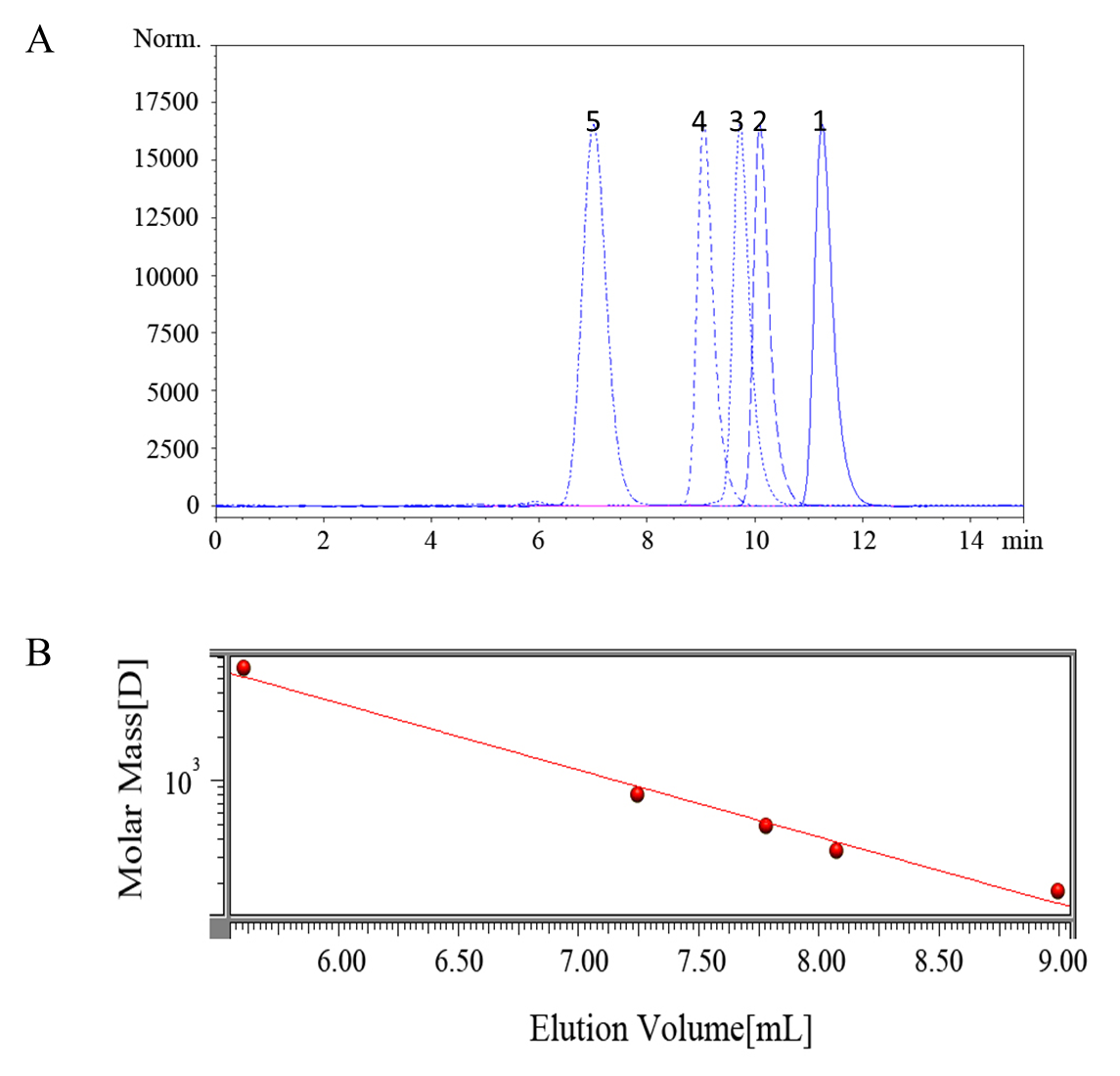


FIGURE S1∣(A) HPLC chromatograms of D-Glucose, sucrose, maltotriose, maltopentaose, polysaccharide standard (molecular weight of 6100 Da). (B) HPGPC standard curve of D-Glu, sucrose, maltotriose, maltopentaose, polysaccharide standard (molecular weight of 6100 Da). Sample was eluted with deionized water at a flow rate of 0.80 mL/min.

Design of extraction conditions

Effect of extraction times on content of DOOS yield

An increasing number of extraction times can enhance the product yield, however, it also means that more input of human and material resources. Thus, it is essential to choose an appropriate number of extraction times. According to **Figure S2A**, content of DOOS yield was higher with the increase of extraction time. The yield of DOOS from the two times extraction (6.47%) increased significantly over a single extraction (4.96%), while the increase in the yield of DOOS from the three times extraction (6.89%) was smaller than that from the two times extraction. Considering the cost of extraction, the two times were selected.

Single factor experiments

Experiments were designed to evaluate the effects of various parameters (extraction temperature, ratio of solution to material and extraction time) on the yields of DOOS. The results of single factor experiments were shown in **Figure S2**.

Effect of extraction temperature on DOOS yield

Extraction temperature potentially has effect on the mass transfer and solubility (7). The extractions were carried out at 50, 60, 70, 80, 90 and 100℃ to investigate the effect of temperature on the yield of DOOS. According to **Figure S2B**, the yield increased and then decreased with increasing temperature, reaching a peak at 70℃ (6.57%). A possible explanation is that the higher solvent diffusion coefficients with lower viscosity at high temperature resulting in higher solubility of oligosaccharides in water, and the enzyme contained in the dried stem of *D. officinale* promotes the release of oligosaccharides, and the optimum temperature of this enzyme is around 70℃ (8).

Effect of ratio of solution to material on DOOS yield

In a certain range, increasing the ratio of solution to material facilitates the complete immersion of the raw material into the liquid and helps to increase the contact surface area between the plant substrate and the solvent, thus increasing the yield of the target compound (9, 10). To investigate the effect of the ratio of solution to material on the yield of DOOS, the ratio of solution to material was set at 10, 20, 30, 40, 50, and 60 mL/g. As shown in **Figure S2C**, the yield of oligosaccharides increased continuously with the increase of the ratio of solution to material. This may be due to the fact that a larger ratio of solution to material is conducive to increasing the contact surface area of the plant matrix with the solvent and the concentration gradient between the solid and liquid phase and the reduced viscosity of the extract, resulting in higher yields of the oligosaccharides.

Effect of extraction time on DOOS yield

Extraction time influences the specific surface area of the solids in contact with the solvent (11). The effect on the DOOS yield of different extraction time from 0.5 to 3 h was displayed in Figure S2D. According to the results shown in **Figure S2D**, the yield of oligosaccharides increased with the increment of extraction time from 0.5h to 2h, and reached the peak value (6.84%). It is speculated that the oligosaccharides need a long enough time to be diffused into the solute. However, excessive heating time may lead to the degradation of oligosaccharides (12).

Based on the results of single-factor experiments, the three conditions with the highest yields were selected for RSM experiments: extraction temperature 70-90℃, ratio of solution to material 40-60 mL/g and extraction time 2-3 h.


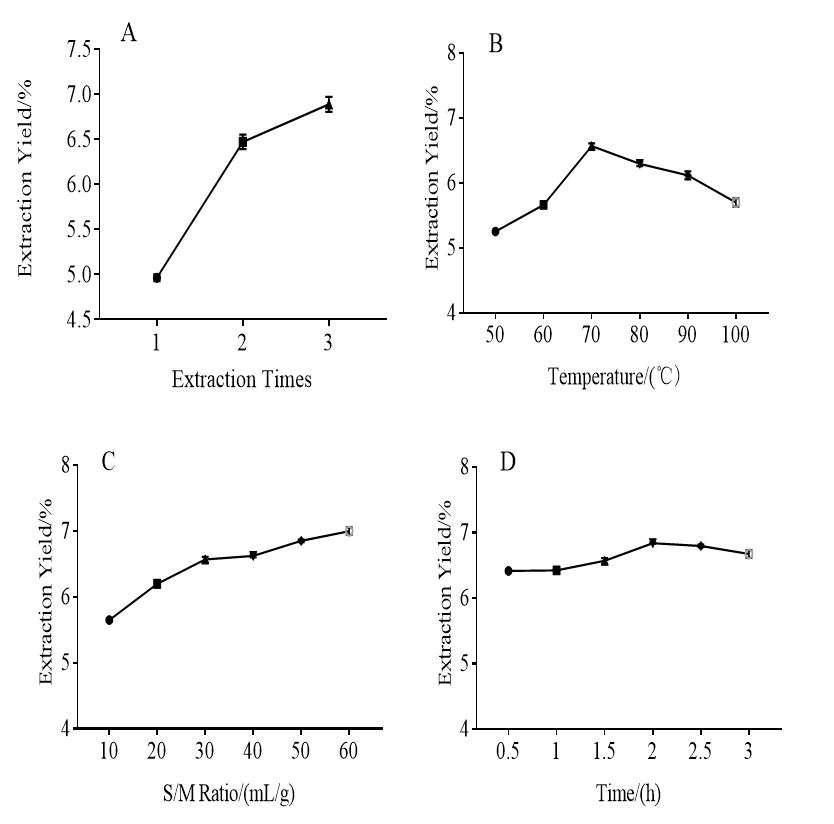


FIGURE S2∣ The effects of extraction times (A), extraction temperature (B), ratio of solution to material (C) and extraction time (D) on the yield of DOOS. Values are the means of DOOS yield (n=3).

Optimization of DOOS extraction by RSM

Statistical analysis and the model fitting

The effect of three individual variables including extraction temperature (X_1_), extraction time (X_2_) and ratio of solution to material (X_3_) on the yield (Y) of DOOS based on RSM are displayed in **Table S2**. The experimental data were analyzed using Design Expert 8.0.5 software, and the results showed that the response variable Y can be expressed by the following quadratic polynomial equation:

Y=6.91-0.15X_1_-0.016X_2_+0.061X_3_+0.013X_1_X_2_-0.037X_1_X_3_+0.075X_2_X_3_+0.22X_1_^2^+0.059X_2_^2^+0.041X_3_^2^ (7)

The results of variance analysis were shown in **Table S3**. The P-value of the model was less than 0.0001, indicating that the regression equation was well fitted. The P-value of the lack of fit was 0.1008 > 0.05, indicating that the model was sufficiently predictive in the range of variables used. The values of determination coefficient (R^2^) and adjusted determination coefficient (R^2^_adj_) were 0.9870 and 0.9755, respectively, indicating that the model fitted the experimental reality well. The coefficient of variation (C.V.) value was 0.42%, indicating that the precision of the model was reliable.

The significance of the effect for each of the variables in the regression equation was evaluated by the P-value of the F-test. As shown in **Table S3,** the variables that had a significant effect on DOOS yield were the linear coefficients (X_1_, X_3_), quadratic coefficients (X_1_^2^, X_2_^2^ and X_3_^2^) and interaction coefficient (X_1_ X_3_, X_2_ X_3_).

Table S2∣Experimental design with response of independent variables

| Run | Independent variables ^1)^ | | | Extraction yield (%) |
| --- | --- | --- | --- | --- |
|  | X_1_ (℃) | X_2_ (h) | X_3_ (mL/g) |  |
| 1 | 70（-1） | 2（-1） | 50（0） | 7.39 |
| 2 | 90（+1） | 2（-1） | 50（0） | 7.05 |
| 3 | 70（-1） | 3（+1） | 50（0） | 7.31 |
| 4 | 90（+1） | 3（+1） | 50（0） | 7.02 |
| 5 | 70（-1） | 2.5（0） | 40（-1） | 7.15 |
| 6 | 90（+1） | 2.5（0） | 40（-1） | 6.95 |
| 7 | 70（-1） | 2.5（0） | 60（+1） | 7.31 |
| 8 | 90（+1） | 2.5（0） | 60（+1） | 6.96 |
| 9 | 80（0） | 2（-1） | 40（-1） | 6.78 |
| 10 | 80（0） | 3（+1） | 40（-1） | 6.92 |
| 11 | 80（0） | 2（-1） | 60（+1） | 7.09 |
| 12 | 80（0） | 3（+1） | 60（+1） | 6.93 |
| 13 | 80（0） | 2.5（0） | 50（0） | 6.89 |
| 14 | 80（0） | 2.5（0） | 50（0） | 6.91 |
| 15 | 80（0） | 2.5（0） | 50（0） | 6.9 |
| 16 | 80（0） | 2.5（0） | 50（0） | 6.94 |
| 17 | 80（0） | 2.5（0） | 50（0） | 6.92 |

1) X_1_^:^ extraction temperature, X_2_: extraction time, X_3_: ratio of solution to material.

Table S3∣ Results of analysis of variance (ANOVA) for the yield of DOOS.

| Source | Sum of squares | df | Mean square | F-value | p-value | Significance^a^ |
| --- | --- | --- | --- | --- | --- | --- |
| Model | 0.47 | 9 | 0.05 | 59.28 | ＜0.0001 | ** |
| X_1_ | 0.17 | 1 | 0.17 | 199.57 | ＜0.0001 | ** |
| X_2_ | 2.11×10^-3^ | 1 | 2.11×10^-3^ | 2.42 | 0.1636 | ns |
| X_3_ | 0.03 | 1 | 0.03 | 34.41 | 0.0006 | ** |
| X_1_ X_2_ | 6.25×10^-4^ | 1 | 6.25×10^-4^ | 0.72 | 0.4252 | ns |
| X_1_ X_3_ | 5.63×10^-3^ | 1 | 5.63×10^-3^ | 6.45 | 0.0387 | * |
| X_2_ X_3_ | 0.02 | 1 | 0.02 | 25.80 | 0.0014 | ** |
| X_1_^2^ | 0.21 | 1 | 0.21 | 236.86 | ＜0.0001 | ** |
| X_2_^2^ | 0.02 | 1 | 0.02 | 16.81 | 0.0046 | ** |
| X_3_^2^ | 7.08×10^-3^ | 1 | 7.08×10^-3^ | 8.12 | 0.0247 | * |
| Residual | 6.11×10^-3^ | 7 | 8.72×10^-4^ |  |  |  |
| Lack of Fit | 4.63×10^-3^ | 3 | 1.54×10^-3^ | 4.17 | 0.1008 | ns |
| Pure Error | 1.48×10^-3^ | 4 | 3.70×10^-4^ |  |  |  |
| Total | 0.47 | 16 |  |  |  |  |
|  | R^2^=0.9870 |  |  |  |  |  |
|  | R_adj_^2^=0.9704 |  |  |  |  |  |
|  | C.V.%=0.42 |  |  |  |  |  |

^a^ * means significant, p < 0.05; ** means highly significant, p < 0.01; ns means not significant.

Analysis of response surfaces

The results of the three-dimensional response surface and two-dimensional contours drawn by Design Expert software were shown in **Figure S3**. Based on Eq. (7), the optimized extraction parameters of the DOOS are as follows: extraction temperature (X_1_) 70℃, extraction time (X_2_) 2 h and ratio of solution to material (X_3_) 60 mL/g by Design Expert software. Experiments were performed three times under these conditions, the yield of DOOS was 7.32 ± 0.03%, which was close to 7.50% predicted by Design Expert software, indicating that the model of Eq. (7) was feasible.


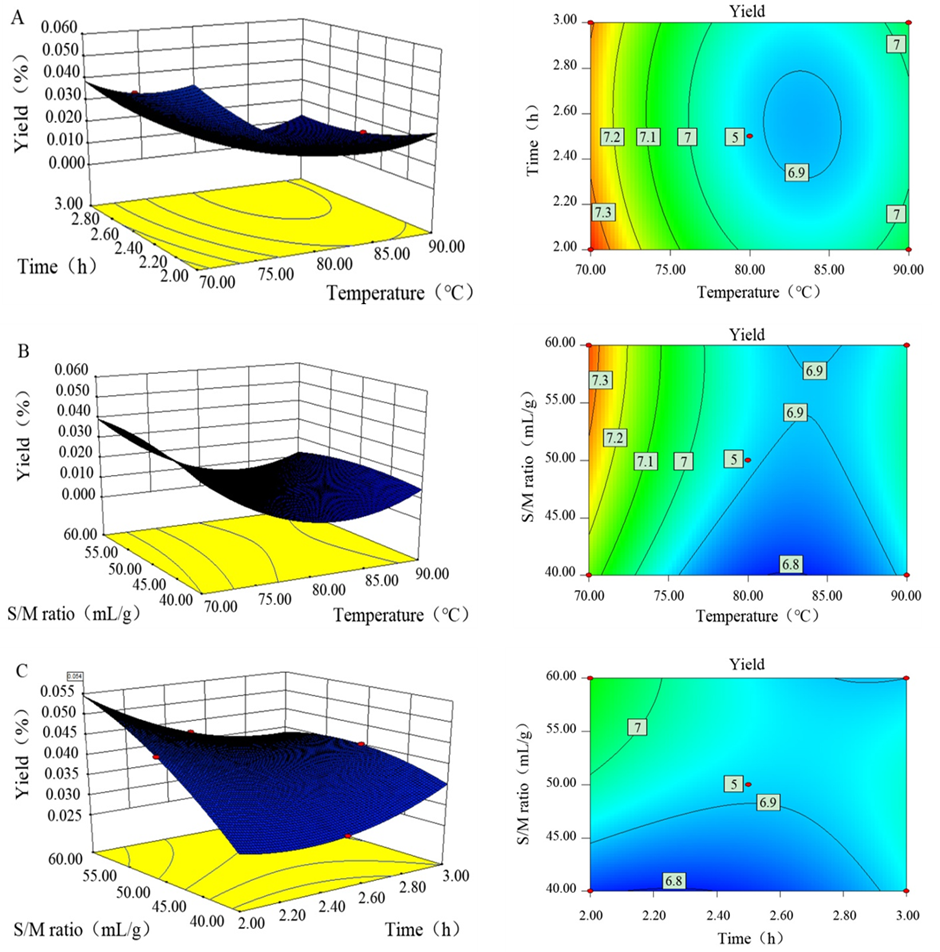


FIGURE S3∣ The interactive effect on the extraction yields of DOOS (left, response surface plots; right, contour plots). (A) Effect of extraction temperature (X_1_) and extraction time (X_2_) on the yield of DOOS; (B) Effect of extraction temperature (X_1_) and S/M ratio (X_3_) on the yield of DOOS; (C) Effect of extraction time (X_2_) and S/M ratio (X_3_) on the yield of DOOS.

Quantitative determination of 48 batches of DOOS

Response surface methodology was performed to extract 48 batches of DOOS and HPLC-RID analysis was applied to obtain the DOOS yields of each batch. According to the results in **Figure S4**, a large variability in the yield of DOOS from different batches can be observed. In terms of origin, DOOS content was higher in batches from Fujian region, and the content of DOOS varied more in batches from Yunnan region, which might be related to the large variance of climate and humidity in different regions of Yunnan. Considering the collection periods, the earlier the batches were collected, the lower the oligosaccharide yields were, indicating that the length of storage time had a significant effect on the oligosaccharide content.


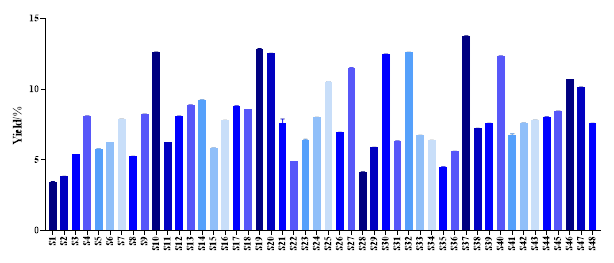


FIGURE S4∣ 48 batches of DOOS yield. Values are the means of DOOS yield (n=3).

Conclusions

In the present study, to improve the extraction yield of DOOS, RSM was successfully used to optimize the extraction process as well as the effects of various factors and their interactions were analyzed. The yield of DOOS was 7.32±0.03% under the optimal extraction conditions (extraction time 2 h, material to water ratio 60 mL/g, extraction temperature 70℃ and extraction two times), which was in good agreement with the predicted value of 7.50%. Under the response surface optimized extraction conditions, the HPLC fingerprint of 48 batches of DOOS samples were developed and analyzed with similarity analysis and hierarchical cluster analysis. The large variation in the similarity of different batches of *D. officinale* indicates that oligosaccharides are susceptible to soil condition, harvesting season, altitude, cultivation environment, and the length of storage time. Our studies results lay a solid foundation for the further quality control studies of *D. officinale*.

Part S2 Tables and Figures

TABLE S4 Raw Samples of *D. officinale* Investigated in This Work

| Sample no. | Source | Specifications | Collecting time |
| --- | --- | --- | --- |
| S1  S2  S3  S4  S5  S6  S7  S8  S9  S10  S11  S12  S13  S14  S15  S16  S17  S18  S19  S20  S21  S22  S23  S24  S25  S26  S27  S28  S29  S30  S31  S32  S33  S34  S35  S36  S37  S38  S39  S40  S41  S42  S43  S44  S45  S46  S47  S48 | Zhejiang  Zhejiang  Zhejiang  Zhejiang  Leqing,Zhejiang  Wuyi,Zhejiang  Wuyi,Zhejiang  Wuyi,Zhejiang  Yandang Mountain, Zhejiang  Yandang Mountain, Zhejiang  Yandang Mountain, Zhejiang  Yandang Mountain, Zhejiang  Yandang Mountain, Zhejiang  Yandang Mountain, Zhejiang  Shouxian Valley, Zhejiang  Fujian  Fujian  Xiamen,Fujian  Xiamen,Fujian  Liantianfu,Fujian  Liantianfu,Fujian  Yunnan  Yunnan  Yunnan  Yunnan  Mangshi,Yunnan  Mangshi,Yunnan  Xishuangbanna, Yunnan  Longling,Yunnan  Longling,Yunnan  Longling,Yunnan  Luchun,Yunnan  Luchun,Yunnan  Gengma,Yunnan  Gengma,Yunnan  Gengma,Yunnan  Gengma,Yunnan  Ruili,Yunnan  Ruili,Yunnan  Hainan  Hainan  Guangxi  Guangxi  Guankang,Shanghai  Anguo,Hebei  Anguo,Hebei  Anguo,Hebei  Reference crude herb | Fresh strip  Fengdou  Fengdou  Fengdou  Fresh strip  Fengdou  Fengdou  Fresh strip  Fengdou  Fengdou  Fresh strip  Fresh strip  Fengdou  Fresh strip  Fresh strip  Fengdou  Fengdou  Fengdou  Fengdou  Fresh strip  Fengdou  Fresh strip  Fresh strip  Fengdou  Fengdou  Fengdou  Fengdou  Fresh strip  Fresh strip  Fresh strip  Fresh strip  Fresh strip  Fengdou  Fresh strip  Fresh strip  Fengdou  Fresh strip  Fresh strip  Fresh strip  Fresh strip  Fresh strip  Fresh strip  Fresh strip  Fengdou  Fengdou  Fengdou  Fengdou  - | Dec-13  Dec-15  Dec-15  Dec-15  Dec-15  Dec-15  Dec-15  Dec-15  Dec-15  Dec-15  Dec-15  Dec-15  Jul-16  Dec-18  Apr-17  Dec-15  Dec-15  Dec-15  Dec-15  Mar-17  Jan-16  May-14  Dec-15  Dec-15  Mar-17  Dec-15  Dec-15  Apr-17  Dec-15  Dec-15  Dec-15  Dec-16  Dec-16  Mar-17  Apr-17  Jan-17  Jan-17  Apr-17  Apr-17  Apr-17  Apr-17  Apr-17  Apr-17  Mar-14  Aug-19  Aug-19  Aug-19  - |

**TABLE S5.** Method validation data: precision, stability, repeatability.

| Peak No. | Precision(n=6) | | Stability(n=6) | | Repeatability(n=6) | |
| --- | --- | --- | --- | --- | --- | --- |
|  | RSD of RRT (%) | RSD of RPA (%) | RSD of RRT (%) | RSD of RPA (%) | RSD of RRT (%) | RSD of RPA (%) |
| 1 | 0.00 | 0.00 | 0.00 | 0.00 | 0.00 | 0.00 |
| 2 | 0.19 | 1.30 | 0.09 | 1.69 | 0.17 | 0.58 |
| 3 | 0.13 | 1.63 | 0.05 | 1.57 | 0.06 | 2.85 |
| 4 | 0.04 | 1.59 | 0.04 | 1.25 | 0.03 | 2.51 |
| 5 | 0.10 | 1.17 | 0.08 | 1.21 | 0.09 | 1.77 |
| 6 | 0.08 | 1.94 | 0.08 | 0.78 | 0.05 | 1.62 |
| 7 | 0.09 | 1.28 | 0.08 | 1.80 | 0.10 | 1.28 |
| 8 | 0.03 | 1.32 | 0.06 | 1.58 | 0.06 | 1.89 |
| 9 | 0.04 | 1.42 | 0.21 | 0.98 | 0.23 | 2.05 |
| 10 | 0.10 | 1.74 | 0.11 | 1.15 | 0.08 | 2.34 |

**TABLE** **S6** The similarities of DOOS from different batches

| No. | Similarity |
| --- | --- |
| S1  S2  S3  S4  S5  S6  S7  S8  S9  S10  S11  S12  S13  S14  S15  S16  S17  S18  S19  S20  S21  S22  S23  S24  S25  S26  S27  S28  S29  S30  S31  S32  S33  S34  S35  S36  S37  S38  S39  S40  S41  S42  S43  S44  S45  S46  S47  S48 | 0.968  0.989  0.873  0.575  0.934  0.985  0.985  0.993  0.787  0.368  0.487  0.416  0.552  0.758  0.97  0.978  0.982  0.912  0.913  0.926  0.983  0.638  0.719  0.565  0.6  0.598  0.222  0.781  0.977  0.995  0.979  0.913  0.947  0.812  0.863  0.725  0.798  0.84  0.899  0.948  0.988  0.975  0.983  0.986  0.291  0.782  0.549  0.961 |


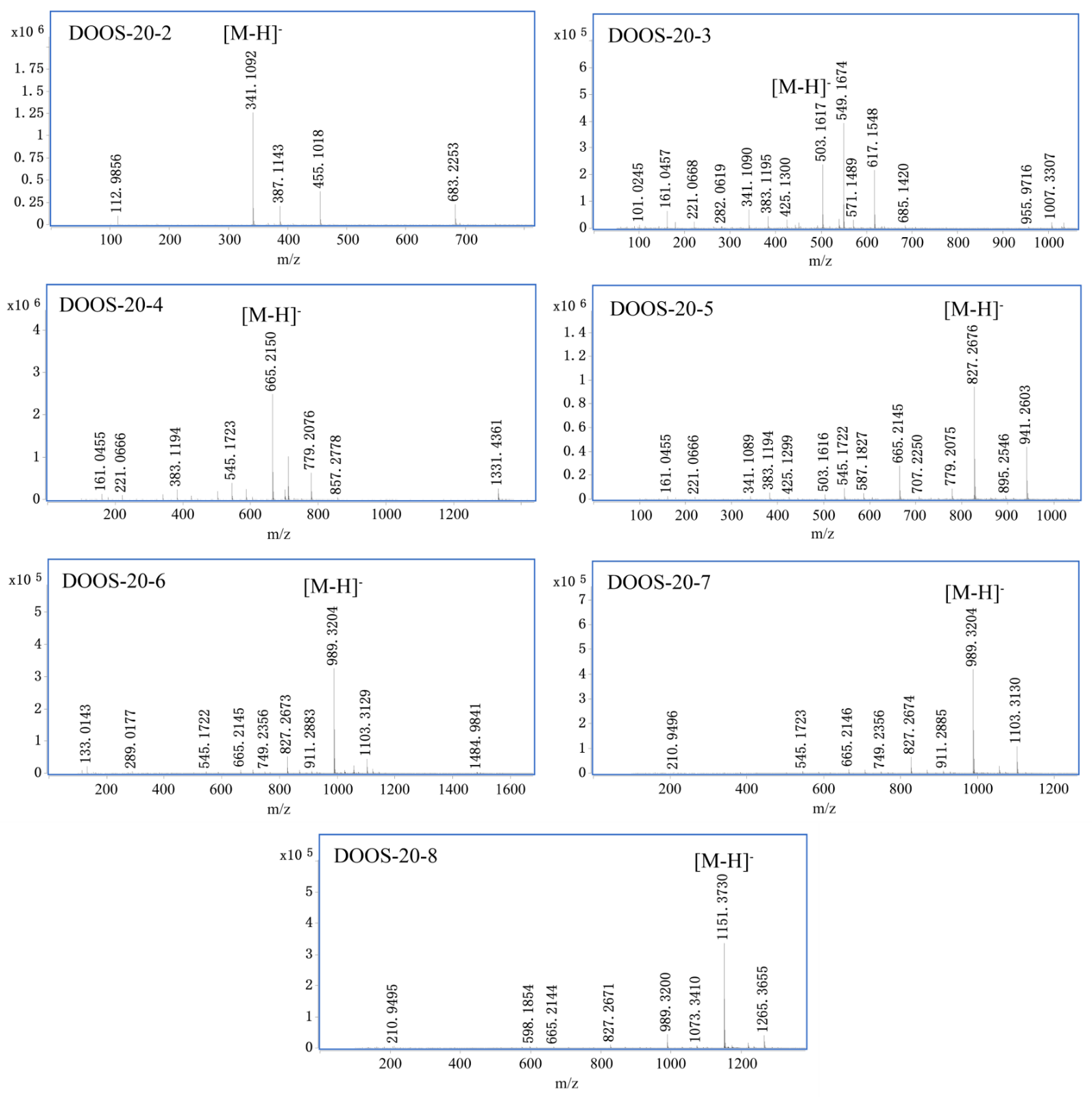


**FIGURE. S5**. HPLC-QTOF-MS analysis of DOOS-20 purified section.


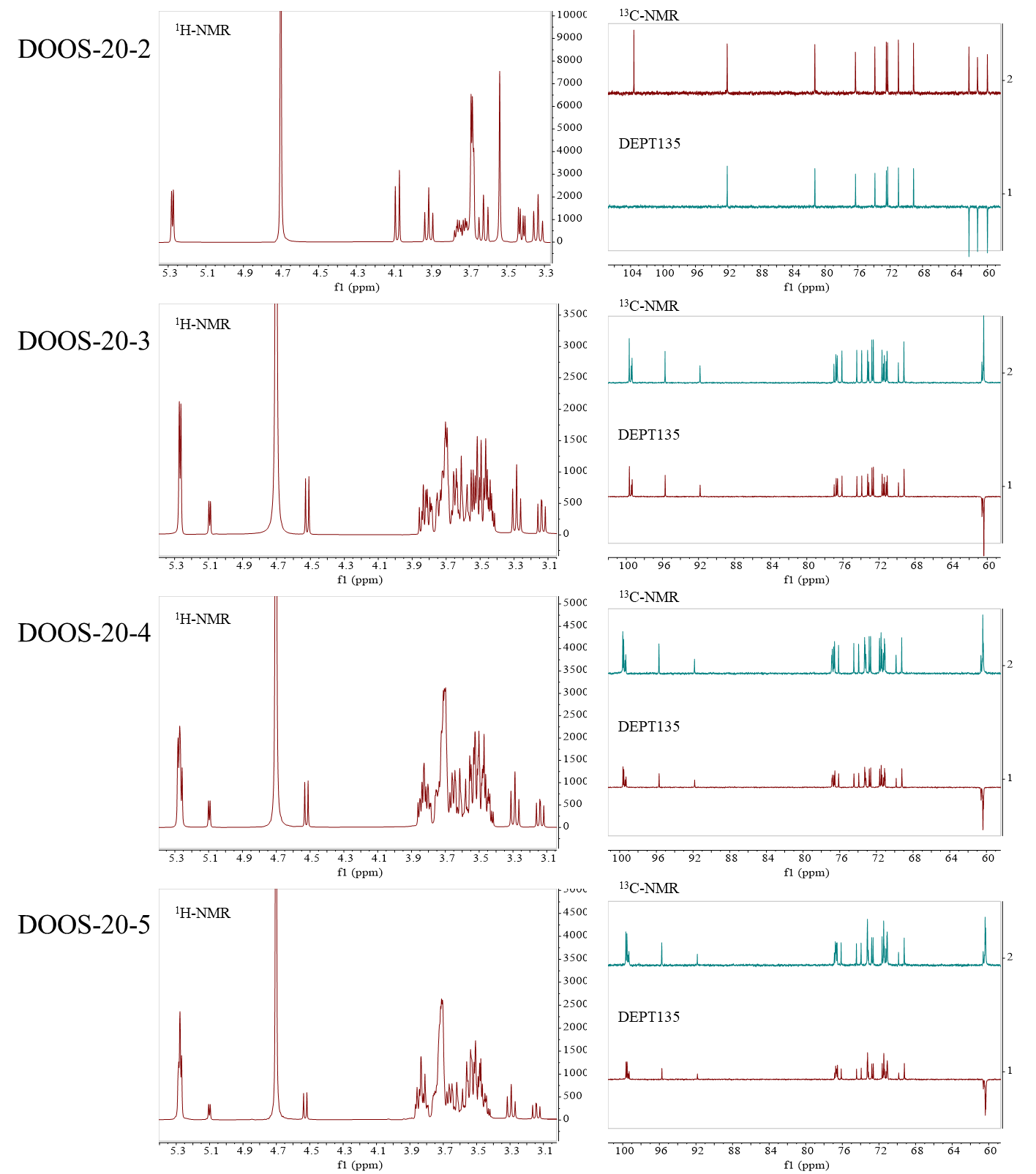


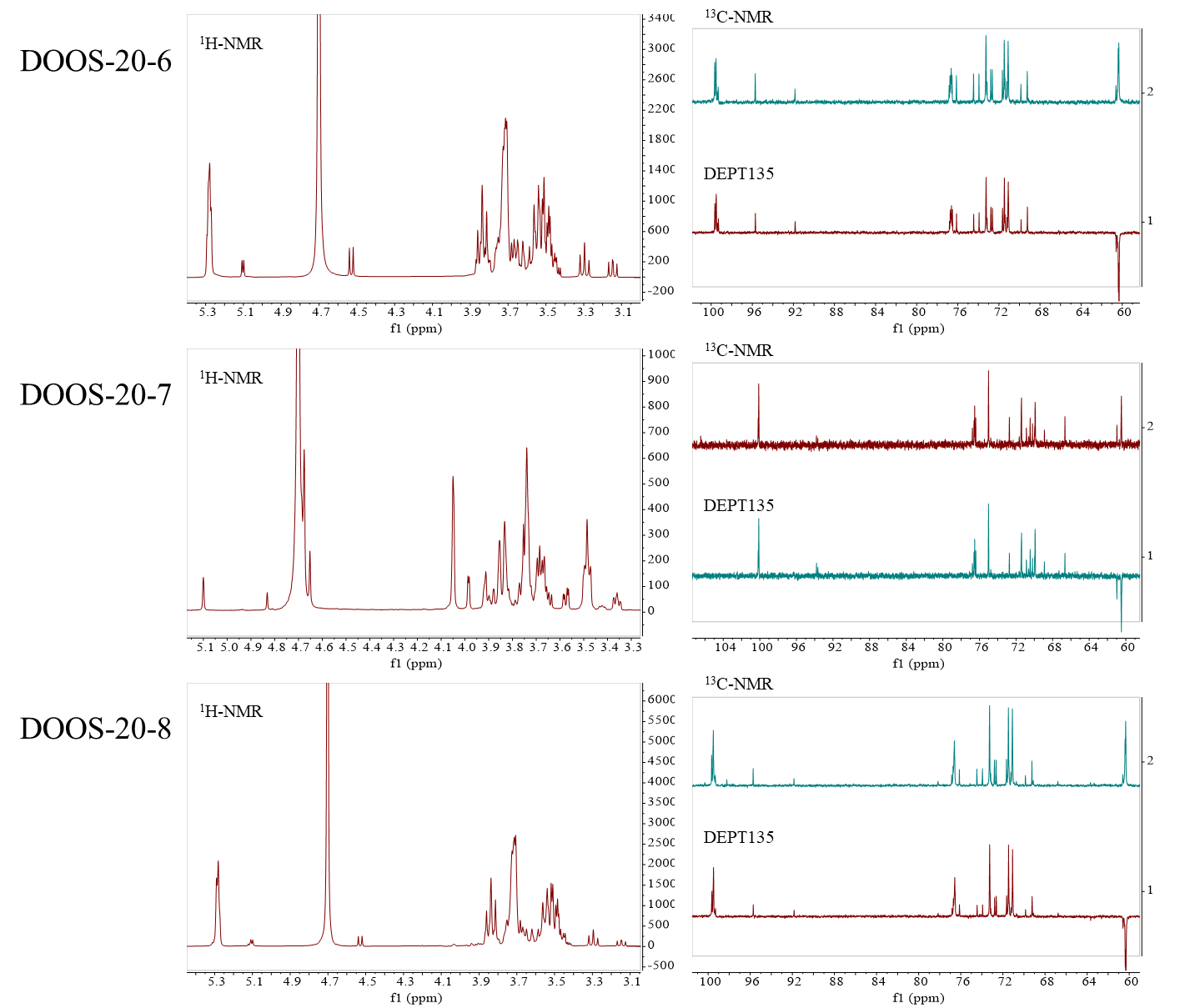


**FIGURE. S6.** ^1^H, ^13^C and DEPT 135 NMR spectra of DOOS-20 purified sections

References

1. L. He, X. Yan, J. Liang, S. Li, H. He, Q. Xiong, X. Lai, S. Hou and S. Huang: Comparison of different extraction methods for polysaccharides from *Dendrobium officinale* stem. *Carbohydrate Polymers*, 198, 101-108 (2018) doi:[10.1016/j.carbpol.2018.06.073](https://doi.org/10.1016/j.carbpol.2018.06.073)

2. V. Briones-Labarca, C. Giovagnoli-Vicuna and R. Canas-Sarazua: Optimization of extraction yield, flavonoids and lycopene from *tomato pulp* by high hydrostatic pressure-assisted extraction. *Food Chemistry*, 278, 751-759 (2019) doi:10.1016/j.foodchem.2018.11.106

3. X. Chen, W. Wang, S. Li, J. Xue, L. Fan, Z. Sheng and Y. Chen: Optimization of ultrasound-assisted extraction of Lingzhi polysaccharides using response surface methodology and its inhibitory effect on cervical cancer cells. *Carbohydrate Polymers*, 80(3), 944-948 (2010) doi:10.1016/j.carbpol.2010.01.010

4. Y. Zhu, Q. Li, G. Mao, Y. Zou, W. Feng, D. Zheng, W. Wang, L. Zhou, T. Zhang, J. Yang, L. Yang and X. Wu: Optimization of enzyme-assisted extraction and characterization of polysaccharides from *Hericium erinaceus*. *Carbohydrate Polymers*, 101, 606-613 (2014) doi:10.1016/j.carbpol.2013.09.099

5. S. Feng, H. Cheng, L. Fu, C. Ding, L. Zhang, R. Yang and Y. Zhou: Ultrasonic-assisted extraction and antioxidant activities of polysaccharides from *Camellia oleifera* leaves. *International Journal of Biological Macromolecules*, 68, 7-12 (2014) doi:10.1016/j.ijbiomac.2014.04.026

6. W. H. Zhang, J. Wu, L. Weng, H. Zhang, J. Zhang and A. Wu: An improved phenol-sulfuric acid method for the determination of carbohydrates in the presence of persulfate. *Carbohydrate Polymers*, 227 (2020) doi:10.1016/j.carbpol.2019.115332

7. X. X. Liu, H. M. Liu, Y. Y. Yan, L. Y. Fan, J. N. Yang, X. D. Wang and G. Y. Qin: Structural characterization and antioxidant activity of polysaccharides extracted from jujube using subcritical water. *Lwt-Food Science and Technology*, 117, 9 (2020) doi:10.1016/j.lwt.2019.108645

8. Y. Liu, G. L. Gong, J. Zhang, S. Y. Jia, F. Li, Y. Y. Wang and S. H. Wu: Response surface optimization of ultrasound-assisted enzymatic extraction polysaccharides from *Lycium barbarum*. *Carbohydrate Polymers*, 110, 278-284 (2014) doi:10.1016/j.carbpol.2014.03.040

9. H. Sun, C. Li, Y. Ni, L. Yao, H. Jiang, X. Ren, Y. Fu and C. Zhao: Ultrasonic/microwave-assisted extraction of polysaccharides from *Camptotheca acuminata* fruits and its antitumor activity. *Carbohydrate Polymers*, 206, 557-564 (2019) doi:10.1016/j.carbpol.2018.11.010

10. C. Zhao, Z. Li, C. Li, L. Yang, L. Yao, Y. Fu, X. He, K. Shi and Z. Lu: Optimized extraction of polysaccharides from *Taxus chinensis* var. mairei fruits and its antitumor activity. *International Journal of Biological Macromolecules*, 75, 192-198 (2015) doi:10.1016/j.ijbiomac.2015.01.043

11. M. Hadidi, A. Ibarz and J. Pagan: Optimisation and kinetic study of the ultrasonic-assisted extraction of total saponins from alfalfa (Medicago sativa) and its bioaccessibility using the response surface methodology. *Food Chemistry*, 309, 8 (2020) doi:10.1016/j.foodchem.2019.125786

12. Y. Qu, C. Li, C. Zhang, R. Zeng and C. Fu: Optimization of infrared-assisted extraction of *Bletilla striata* polysaccharides based on response surface methodology and their antioxidant activities. *Carbohydrate Polymers*, 148, 345-353 (2016) doi:10.1016/j.carbpol.2016.04.081
